# WUSCHEL-related homeobox1 (WOX1) regulates vein patterning and leaf size in *Cucumis sativus*

**DOI:** 10.1038/s41438-020-00404-y

**Published:** 2020-11-01

**Authors:** Hu Wang, Huanhuan Niu, Chuang Li, Guoyan Shen, Xiaofeng Liu, Yiqun Weng, Tao Wu, Zheng Li

**Affiliations:** 1 State Key Laboratory of Crop Stress Biology in Arid Areas, College of Horticulture, Northwest A&F University, Yangling, Shaanxi 712100 China; 2 Department of Vegetable Sciences, Beijing Key Laboratory of Growth and Developmental Regulation for Protected Vegetable Crops, China Agricultural University, Beijing, 100193 China; 3 USDA-ARS, Vegetable Crops Research Unit, Horticulture Department, University of Wisconsin, Madison, WI 53706 USA; 4 College of Horticulture and Landscape Architecture, Hunan Agricultural University, Changsha, Hunan 410128 China

**Keywords:** Non-model organisms, Transgenic organisms

## Abstract

In plants, *WUSCHEL-related homeobox1* (*WOX1*) homologs promote lamina mediolateral outgrowth. However, the downstream components linking WOX1 and lamina development remain unclear. In this study, we revealed the roles of WOX1 in palmate leaf expansion in cucumber (*Cucumis sativus*). A cucumber *mango fruit* (*mf*) mutant, resulting from truncation of a WOX1-type protein (CsWOX1), displayed abnormal lamina growth and defects in the development of secondary and smaller veins. *CsWOX1* was expressed in the middle mesophyll and leaf margins and rescued defects of the *Arabidopsis wox1 prs* double mutant. Transcriptomic analysis revealed that genes involved in auxin polar transport and auxin response were highly associated with leaf development. Analysis of the cucumber *mf rl* (*round leaf*) double mutant revealed that CsWOX1 functioned in vein development via PINOID (CsPID1)-controlled auxin transport. Overexpression of *CsWOX1* in cucumber (*CsWOX1*-OE) affected vein patterning and produced ‘butterfly-shaped’ leaves. CsWOX1 physically interacted with CsTCP4a, which may account for the abnormal lamina development in the *mf* mutant line and the smaller leaves in the *CsWOX1*-OE plants. Our findings demonstrated that CsWOX1 regulates cucumber leaf vein development by modulating auxin polar transport; moreover, CsWOX1 regulates leaf size by controlling *CIN-TCP* genes.

## Introduction

Leaves of higher plants showing adaxial–abaxial patterning originate from dome-shaped leaf primordia^1^. After initiation from the shoot apical meristem (SAM), leaf primordia develop along three axes of asymmetry, a proximal–distal axis, an adaxial–abaxial axis, and a mediolateral axis, to form a flat leaf. The formation of the mediolateral axis (from midrib to margin), which is dependent on adaxial–abaxial polarity and activity of the leaf meristems, promotes leaf expansion^2^. During early leaf development, active cell division occurs in the marginal and submarginal regions of the leaf to ensure normal laminar initiation. Then, cells in this region cease to divide, and cell expansion and differentiation cause lamina expansion^3^.

In *Arabidopsis*, laminar expansion specifically along the mediolateral axis is regulated by a subgroup of *WUSCHEL-RELATED HOMEOBOX* (*WOX*) genes containing *PRESSED FLOWERS* (*PRS*) and *MAEWEST/WOX1*^4,5^. *WOX1* is expressed along with the adaxial–abaxial juxtaposition, overlapping that of *PRS* at the leaf margins^5^. *PRS* and *WOX1* redundantly promote lateral lamina outgrowth, and the *wox1 prs* double mutant produces narrow leaves with disturbed polarity but unaltered leaf length^5^. Similar phenotypes are also associated with mutations of *WOX1* homologs in other species; for example, *narrow sheath* (*ns*) in *Zea mays* L.^6^, *maewest* (*maw*) in *Petunia x hybrida*^4^, *lam1* in *Nicotiana sylvestris*^7^, *stenofolia* (*stf*) in *Medicago truncatula*^7^, and *lath* in *Pisum sativum* L.^8^, all of which show narrow leaf blades and similar expression patterns of *WOX1* homologs, but the leaf length is virtually unaffected.

In *Arabidopsis*, a ‘middle domain’ exists at the junction between the adaxial and abaxial sides of leaves, and auxin mediates the limitation of the middle domain by adaxial and abaxial sides^5,9^. Auxin response activator *MP* (*MONOPTEROS*, also known as *Auxin Response Factor 5* [*ARF5*]) is expressed in the adaxial and middle domains, while the auxin response repressor *ETT* (*ETTIN*, also known as *ARF3*), *ARF2* and *ARF4* are expressed in the abaxial domain^10^. The abaxial auxin maximum, together with region-specific auxin response activators and repressors, activates the expression of *WOX1* and *PRS* in the middle domain of the leaf margin^3^. In addition to the auxin response, polar auxin transport, mediated by auxin efflux carriers (*PIN-FORMED*, *PIN*) and influx carriers (*AUXIN1/LIKE AUX1*, *AUX/LAX*), plays critical roles in organ initiation and morphogenesis by forming an auxin maximum and auxin gradient^11^. Mutation in *PIN1* results in a decreased leaf number, defects in leaf vein pattern, and a distorted leaf shape^12,13^. In *Arabidopsis*, the auxin influx carrier LAX2 controls vasculature formation^14^, and the quadruple mutant *aux1 lax1 lax2 lax3* has fewer and less-dense vascular bundles than the wild type (WT)^15^. *PINOID* (*PID*), encoding a serine/threonine protein kinase, is a positive regulator of auxin polar transport via phosphorylation and polar localization of PIN proteins^16^. In *Arabidopsis*, the *pid* mutant has pleiotropic shoot phenotypes that include defects in the formation of cotyledons and floral organs, which are similar to those of the *pin1* mutant and WT plants treated with auxin-transport inhibitors^16,17^. In addition, overexpression of *OsPID* caused delayed adventitious root formation and curled shoot growth with gravitropism in rice^18^. In pea (*P. sativum*), *PsPK2*, a *PINOID-like* gene, was shown to be involved in the formation of compound leaf^19^. Therefore, despite a conserved role in auxin polar transport, different *PID* homologs participate in distinct developmental processes in different species.

After leaf primordium initiation, cell proliferation and differentiation occur throughout the entire region of the leaf blade^20^. Class II TEOSINTE BRANCHED1/CYCLOIDEA/PROLIFERATING CELL FACTOR (TCP) transcription factors repress marginal meristem activity, thereby promoting the transition from cell proliferation to cell differentiation^21^. Inactivation of TCPs in *Arabidopsis* and inactivation of the orthologs *CINCINNATA* (*CIN*) in *Antirrhinum majus* L. and *LANCEOLATE* (*LA*) in *Solanum lycopersicum* result in cell overproliferation at the leaf margin, leading to expanded, crinkly leaves or leaflets with serrated margins^21,22^. In *Arabidopsis tcp* mutants, the indeterminate proliferation of leaf margin cells is likely due to prolonged activity of WOX1 and PRS, as well as sustained mitotic activity caused by the upregulated expression of *CYCLINB1*^23,24^. Expression of class II *TCPs* is restricted to the distal leaf regions via post-transcriptional repression by miR319^22,25^. Moreover, mutations that disturb this regulation result in smaller leaves with precocious differentiation of the leaf margins and loss of leaflet generation, which is likely due to the ectopic expression of *TCP* transcription factors in the proximal leaf regions^22,26^. Consistent with this finding, TCP transcription factors redundantly inhibit *WOX* expression, terminating the marginal meristem in *Arabidopsis*^23^.

Extensive molecular genetic studies have identified the regulatory network of leaf expansion; however, most focus on *Arabidopsis* leaves with pinnate veins. The analysis of the formation mechanism of palmate veins in cucumber leaves will help explain the diversity of leaf morphogenesis in other species with different leaf vein patterns. In addition, the downstream components linking WOX1 and lamina development need to be explored in other species. In this study, we found that CsWOX1 can regulate palmate vein patterns by modulating PINOID-controlled auxin polar transport and stabilizes leaf size by controlling the CIN-TCP genes at the transcriptional and protein levels. These findings may provide a new understanding of the diversity of leaf morphogenesis in different species.

## Results

### The *mango fruit* mutant exhibits defective leaf growth

**Fig. 1:**
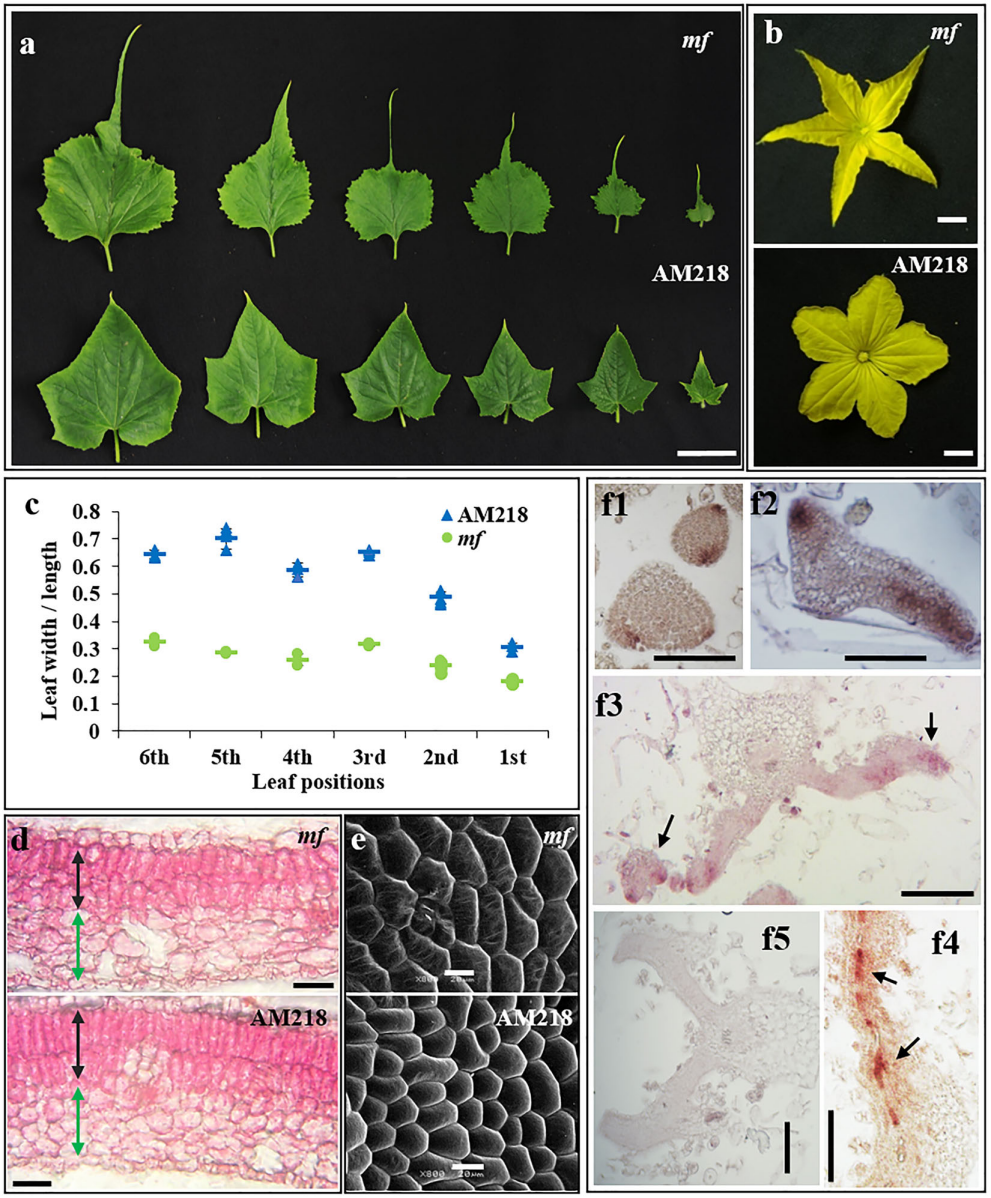
Characterization of the cucumber *WOX1* gene (*CsWOX1*) in leaf and petal tissues. **a** Leaves from the mutant line (*mango fruit*, *mf*) and its corresponding WT line (AM218). Six successive leaves (from the right) were harvested from the top of the same plant. **b** Male flowers of the *mf* mutant (top) and AM218 (bottom). **c** The ratio of average leaf width to length in six successive top leaves from the same plant representing different growth stages. Each point represents a value in the set of data, and the short line represents the mean of this set of data. Five biological repeats were included per measurement. **d** Longitudinal section of a fully expanded second true leaf blade from *mf* and AM218. Black and green arrows indicate palisade mesophyll and spongy mesophyll cells, respectively. **e** Scanning electron microscopy images of a mature leaf adaxial surface in the middle of the lamina from *mf* and AM218. **f** mRNA in situ hybridization analysis of *CsWOX1* expression in developing leaves of AM218. *CsWOX1* transcripts were detected at the early stage of leaf primordia (f1 and f2), the leaf margins (f3, black arrows), and the middle mesophyll layer (f4, black arrows). Sense probe analyses of the leaf (f5) as a negative control. Bar = 5 cm (**a**), 1 cm (**b**), 100 µm (**d**), and 20 µm (**e**, **f**)

In cucumbers, leaf shape is usually palmate, with five primary veins extending from the petiole to five tips on the leaf margin (Fig. 1a)^27^. We previously identified the cucumber mutant *mango fruit* (*mf*), which displays multiple flower growth defects as well as male and female sterility^28^. Map-based cloning revealed that a truncated protein lacking the conserved WUS box of WOX1-type transcriptional regulator (CsWOX1) was responsible for the mutant phenotype^28^. As shown in Fig. 1a, b, the *mf* mutant also showed significant defects in mediolateral axis growth of the true leaves and petals. A comparison of the corresponding successive leaves showed that the *mf* leaves became narrower than the WT (AM218) leaves, and the ratio of leaf width to length in the *mf* leaves was significantly smaller than that in the AM218 leaves (Fig. 1c). The average width of the leaf distal region (from the midpoint of midvein to tip) was measured, and it decreased sharply in *mf* compared with AM218. The width ratio of *mf*/AM218 in the leaf distal region decreased from one-fourth at the 6th leaf position to one-tenth at the 1st leaf position (Supplemental Fig. S1). Moreover, analysis of leaf tissue sections showed loose palisade mesophyll and spongy mesophyll cells and irregular subepidermal cells compared with those of the AM218 leaves (Fig. 1d). Further observations with scanning electron microscopy (SEM) revealed that the leaf epidermal cells were less smooth and were larger in the *mf* leaves than in the AM218 leaves (Fig. 1e; Supplemental Fig. S2). mRNA in situ hybridization (ISH) analysis in AM218 revealed the expression of *CsWOX1* in the early stage of leaf primordia (Fig. 1, f1 and f2) and the middle mesophyll and the leaf margin of the leaf blade (Fig. 1, f3 and f4), similar to the domains of *WOX1* and *PRS* in *Arabidopsis*^4,5,29^.

### CsWOX1 can rescue defects of the *wox1 prs* double mutant of *Arabidopsis*

**Fig. 2:**
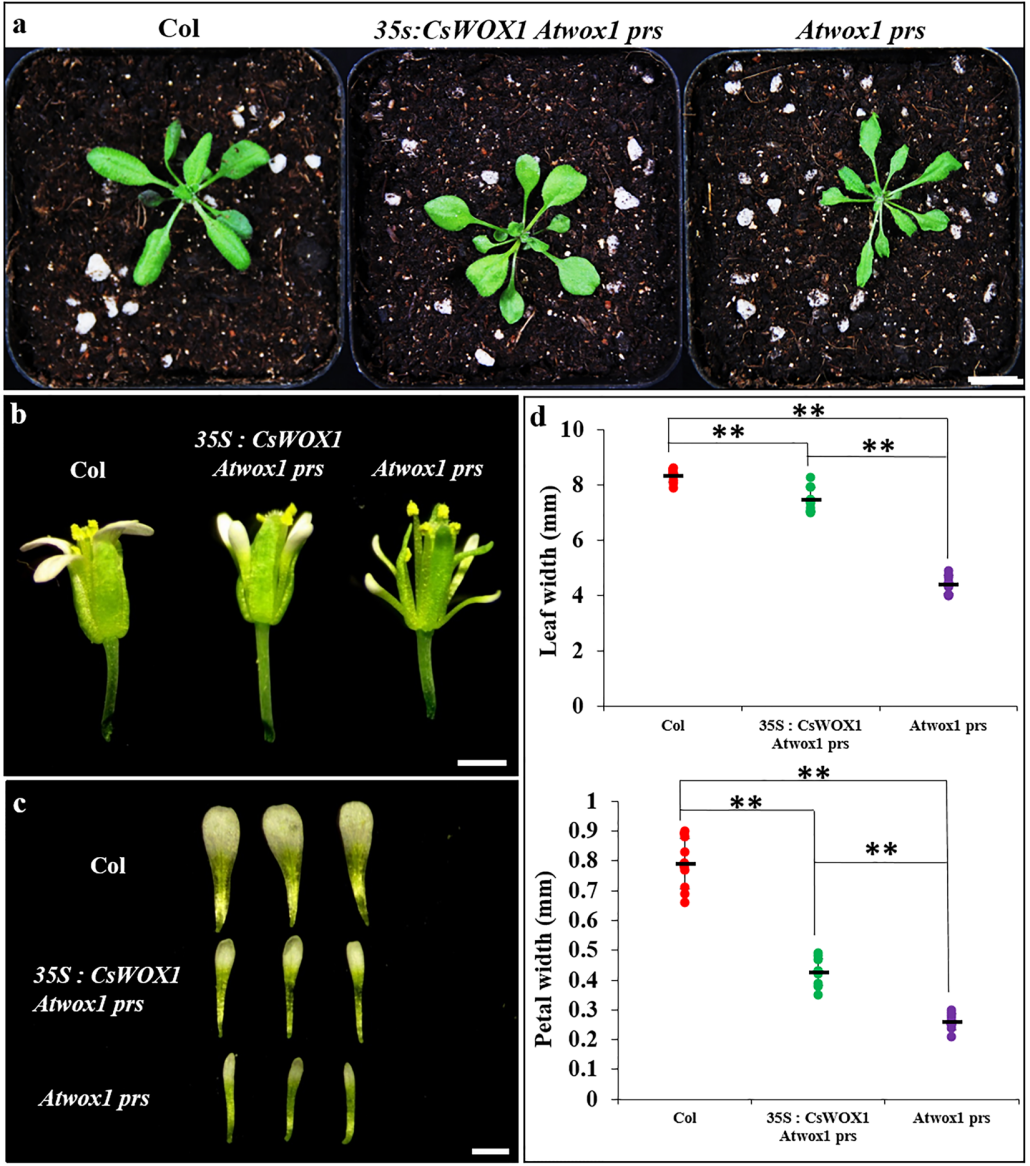
Complementation assay of *CsWOX1* in *Arabidopsis wox1 prs* double mutants. **a** Aerial view of three different *Arabidopsis* seedlings. The *Arabidopsis wox1 prs* (*Atwox1 prs*) double mutant was obtained by the CRISPR-Cas9 method, and overexpression of *CsWOX1* in this double mutant produced the *35S:CsWOX1 Atwox1 prs* lines. The narrower leaves of the *wox1 prs* double mutant were rescued by overexpression of *CsWOX1*. **b** Flowers from the WT, *35S:CsWOX1 Atwox1 prs* line, and *wox1 prs* double mutant. **c** Individual petals from the flowers shown in (**b**). The narrower petals of the *wox1 prs* double mutant were also rescued by overexpression of *CsWOX1*. **d** Measurements of the leaf (top) and petal (bottom) width in the WT, *35S:CsWOX1 Atwox1 prs* line, and *wox1 prs* double mutant. The sixth fully expanded rosette and flowers in the top inflorescence of the main stem were used to measure the width. Each point represents a value in from the dataset, and the short black line represents the mean of this set of data. Ten biological repeats were included per measurement. Significance tests were performed using Student’s *t*-test at the 0.05 and 0.01 levels (*p-value <0.05; **p-value <0.01). *Arabidopsis* Columbia ecotype (Col) was used as the wild type (WT) in all assays. Bar = 1 cm (**a**) and 1 mm (**b**, **c**)

Previous studies have revealed the narrow leaf phenotype of the *wox1 prs* double mutant in *Arabidopsis*. We performed knockout of both *WOX1* and *PRS* in *Arabidopsis* (Fig. 2; Supplemental Fig. S3). True leaves and flower petals of the *wox1 prs* double mutant from four independent lines showed a significant reduction in blade expansion (Fig. 2a–c). To determine whether the function of *WOX1* is conserved in cucumber and *Arabidopsis*, we ectopically expressed *CsWOX1* in the *wox1 prs* double mutant. The transgenic lines showed an obvious rescue of the leaf extension defect caused by loss-of-function of *WOX1* and *PRS* (Fig. 2). True leaves and flower petals of the complemented plants became obviously wider than those of the *wox1 prs* double mutant, and the leaves exhibited improved restoration compared to the flower petals (Fig. 2d).

### Transcriptome profiling reveals that auxin participates in *CsWOX1*-related leaf development in cucumber

Young leaves (~1 cm) in the apical bud of the AM218 and *mf* plants were used to perform transcriptomic analysis with RNA-Seq. A total of 224 genes were differentially expressed between AM218 and *mf*. Of these, 163 were upregulated and 61 were downregulated in the leaves of the *mf* plants (Supplemental Table S1). Notably, certain genes related to organ development were significantly downregulated in the *mf* leaves, including *Csa3G895620* from the leucine-rich receptor-like protein kinase family, *Csa4G043850* from the homeobox-leucine zipper protein family, and *Csa6G095280* from the MADS-box family (Agamous-like MADS-box protein) (Supplemental Table S2). In addition, a total of seven auxin-related genes, including genes involved in polar auxin transport and auxin response, were differentially expressed between the *mf* and AM218 leaves (Table 1). The peptidyl-prolyl *cis/trans* isomerase *Pin1At* (*Csa4G046790*) displayed a 2.04-fold (log_2_FC = 1.03) increase in the *mf* mutant. Pin1At affects auxin transport and polar localization of PIN1 via PID and Protein Phosphatase 2A (PP2A) mediation^30^. Furthermore, Pin1At catalyzes the conformational change in phosphorylated Ser/Thr-Pro motifs of PIN1^30^. In addition, the *ABCB* gene (*Csa5G585950*) displayed a 2.05-fold (log_2_FC = 1.03) increase in the *mf* mutant. The *ABCB* gene family encodes phosphor glycol proteins (PGPs) belonging to the ATP-binding cassette subfamily B type transporters, which were shown to participate in auxin efflux via stabilization of PIN proteins^31,32^. In contrast, the auxin transporter-like protein gene *LAX4* (*Csa2G264590*), which encodes a member of the AUX/LAX family of auxin influx carriers, displayed a 78% (log_2_FC = −2.16) decrease in the *mf* mutant. *LAX2* controls vasculature development in *Arabidopsis*, and deficiency of the *LAX2* gene resulted in vascular breaks in the cotyledons^14^. In addition, *AUX28-LIKE* (*Csa2G225320*), which was predicted as an auxin-inducible protein, displayed a 95% (log_2_FC = −4.19) decrease in the *mf* leaves. These data suggest that the transcription levels of auxin-transport-related and leaf development genes are disturbed in the *mf* mutant.

**Table 1:** Differentially expressed auxin-related genes in RNA-Seq analysis of the *mf* mutant and wild type (AM218)

Gene ID	log_2_FC	FDR	Putative function in CuGenDB
*Csa2G059200*	1.19	3.09E−02	Auxin-responsive protein, IAA33-like
*Csa2G225320*	−4.19	3.31E−08	Auxin-induced protein, AUX28-like
*Csa2G264590*	−2.16	2.89E−03	Auxin influx carriers, LAX4
*Csa6G485160*	1.53	1.53E−03	Auxin-induced protein, 5NG4-like
*Csa7G398090*	1.11	4.51E−02	Auxin-induced protein, ARG2-like
*Csa4G046790*	1.03	1.40E−06	Peptidyl-prolyl *cis*–*trans* isomerase, PIN1At
*Csa5G585950*	1.03	1.80E−06	Auxin efflux transporter, ABCB4-like

### The regulatory effect of CsWOX1 on the development of the leaf vein is associated with CsPID1

**Fig. 3:**
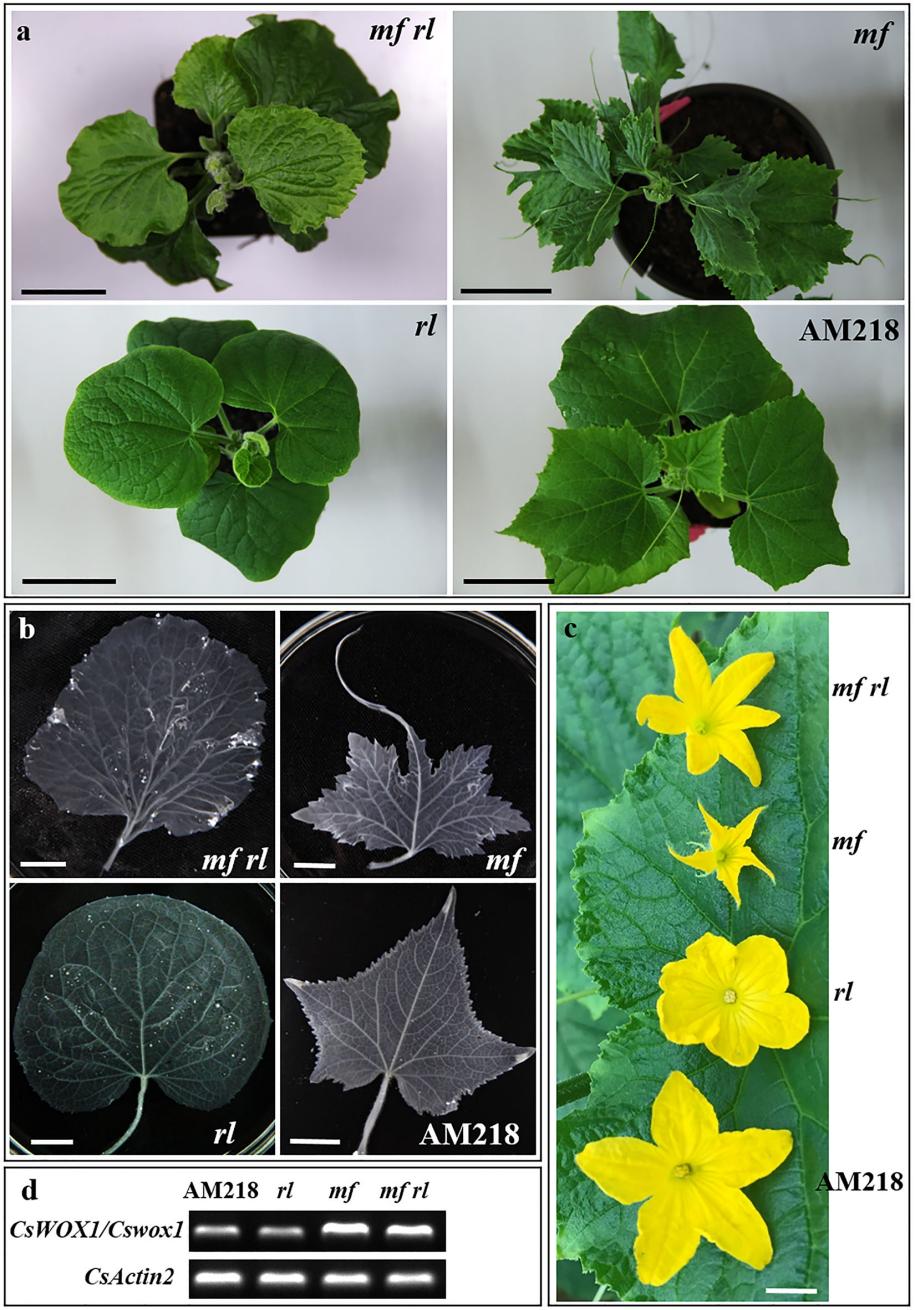
Phenotypic characterization of the cucumber *mf* mutant, *round leaf (rl)* single mutant, and *mf rl* double mutant. **a** Aerial view of all three mutants and wild-type AM218. **b** The patterning and distribution of veins in the three mutants and AM218. **c** The male flowers in the three mutants and AM218. **d** Semiquantitative PCR assay of *CsWOX1*/*Cswox1* expression in different plants. Bar = 4 cm (**a**) and 1 cm (**b**, **c**)

The cucumber *round leaf* (*rl*) mutant^33^, which is the result of a nonsynonymous single nucleotide polymorphism (SNP) in the gene encoding the protein kinase PINOID, has round leaves with a smooth margin and disturbed leaf venation (Fig. 3a). Rather than extending from the petiole to the leaf margin, the five primary veins in the *rl* mutant branched repeatedly into secondary and smaller veins before reaching the leaf margin (Fig. 3a, b)^33^. In the *mf* mutant, the midvein grew normally, but growth of the secondary veins at the distal part of the leaf blade was inhibited (Fig. 3a, b). This decrease in number and interrupted extension of secondary and minor veins resulted in a markedly different vein pattern in *mf* relative to AM218 (Fig. 3b).

Heterozygous individuals of the *mf* and *rl* mutant genes were subsequently used for hybridization. The hybrid progeny with two heterozygous loci were self-pollinated to generate the *mf rl* double mutant with the two recessive homozygous loci (Fig. 3a). The *mf rl* double mutant showed a similar leaf shape to the *rl* mutant, with the primary veins branching to produce secondary veins before reaching the leaf margin (Fig. 3b). The *mf rl* double mutant also reduced the severity of defects in petal shape, with wider petals than those in the *mf* plants (Fig. 3c). Transcription analysis indicated that *CsWOX1* expression was not obviously changed in the *rl* mutant compared with AM218 (Fig. 3d). In addition, the transcription level of the *Cswox1* gene in the *mf rl* double mutants was similar to that in the *mf* mutant (Fig. 3d). However, the expression of *CsPID1* was reduced in the *mf* plants compared with the AM218 plants (shown below in Fig. 5a). Therefore, CsWOX1 may maintain the normal transcriptional expression of *CsPID1*. Overall, these findings suggest that loss-of-function of the protein kinase CsPID1 reduces the severity of leaf shape defects in the *mf* mutant, even though the transcription level of *CsPID1* was decreased in the *mf* leaves.

**Fig. 4:**
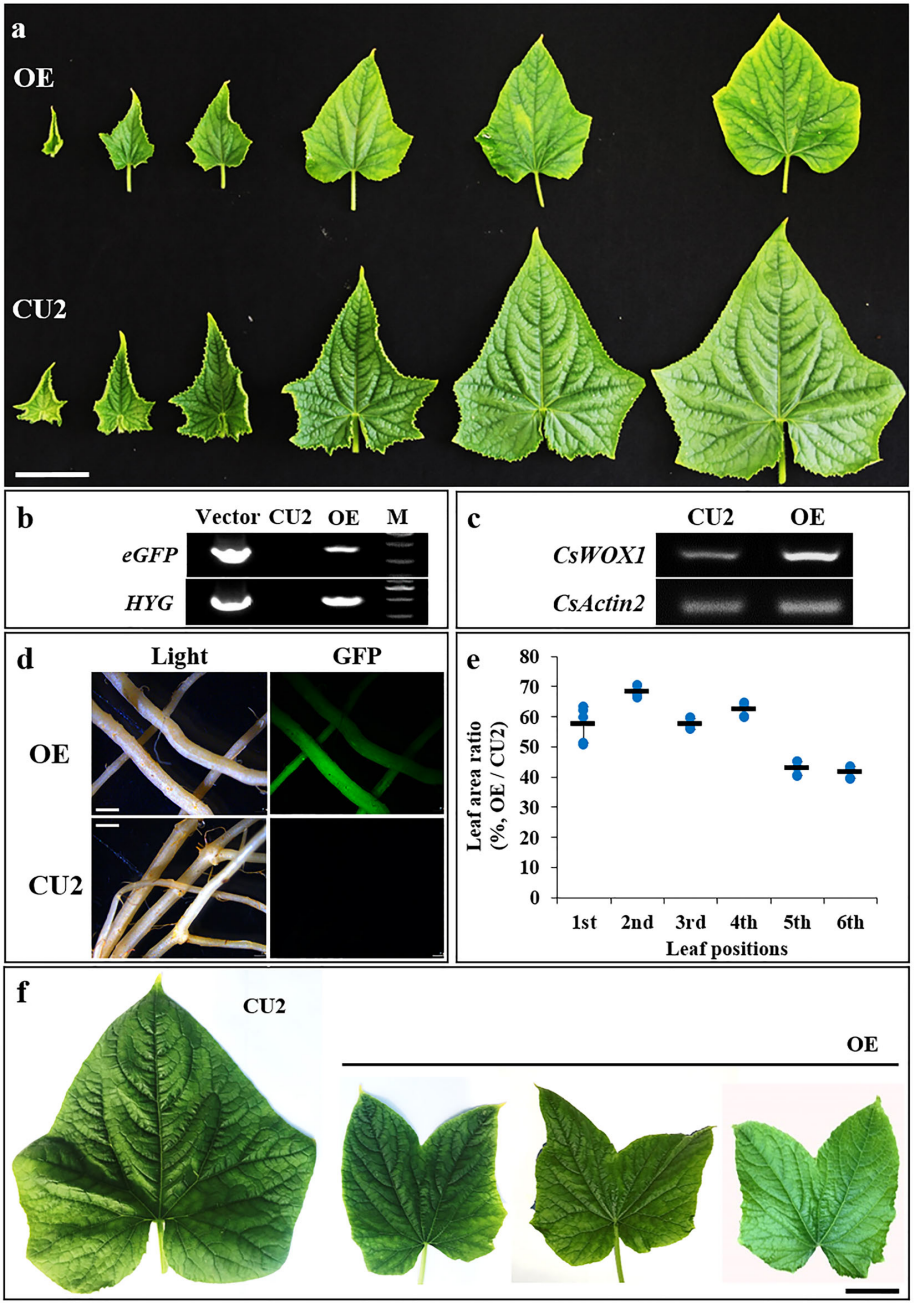
Overexpression of *CsWOX1* (OE) in cucumber. **a** Leaf shape in OE and wild-type CU2. Six successive leaves (from the left) were harvested from the top of the same plant. **b** PCR assay of the OE and CU2 lines. PCR targeted the CDS of the *eGFP* and *HYP* genes in the plasmid. M: DNA marker. **c** Semiquantitative PCR assay of the OE and CU2 leaves. **d** Fluorescence detection of the OE and CU2 roots. **e** The area ratio of leaves from OE to those from CU2 using six successive top leaves from the same plant representing different growth stages. Each point represents a value in the set of data, and the short black line represents the mean of this set of data. Five biological repeats were included per measurement. **f** Abnormal leaves (‘butterfly-shaped’) with seven primary veins extending from the petiole in the OE plant. Bar = 5 cm (**a**), 2 mm (**d**), and 3 cm (**f**)

### Overexpression of *CsWOX1* results in decreased leaf size in cucumber

To determine the functions of *CsWOX1* in cucumber leaf development, we introduced a vector containing a 35S promoter and coding sequence (CDS) of *CsWOX1* into the cucumber CU2 line to generate the overexpression line (*CsWOX1*-OE) (Fig. 4a). The T-DNA domain of the overexpression vector in the transgenic line was identified using PCR amplification based on the hygromycin (*HYG*) and enhanced green fluorescent protein (*eGFP*) genes (Fig. 4b). Expression of *CsWOX1* was upregulated under the control of the 35S promoter in *CsWOX1*-OE (Fig. 4c). The positive transgenic plants were also confirmed by the GFP fluorescence observed in the roots (Fig. 4d). In the *CsWOX1*-OE line, leaf size was obviously smaller than that in the WT (CU2) (Fig. 4a). The leaf area ratio (*CsWOX1*-OE/CU2) of the 1st to 4th leaves from the top was ~60%, while that ratio of the relatively mature blade (5th and 6th leaves) was ~45% (Fig. 4e). In CU2, five primary veins extending from the petiole to five tips on the leaf margin and the middle vein, as an axis of symmetry, formed the maximum elongation of all veins (Fig. 3b). However, some abnormalities in the *CsWOX1*-OE leaves were also observed. In ~10% of all leaves, a total of seven primary veins extended from the petiole, and the middle vein, as an axis of symmetry, had the shortest length, accompanied by ‘butterfly-shaped’ leaves (Fig. 4f). The two symmetrical parts contained a long ‘middle vein’, which formed the furthest tips, and this ‘middle vein’ was also attached to symmetrical secondary veins (Fig. 4f).

**Fig. 5:**
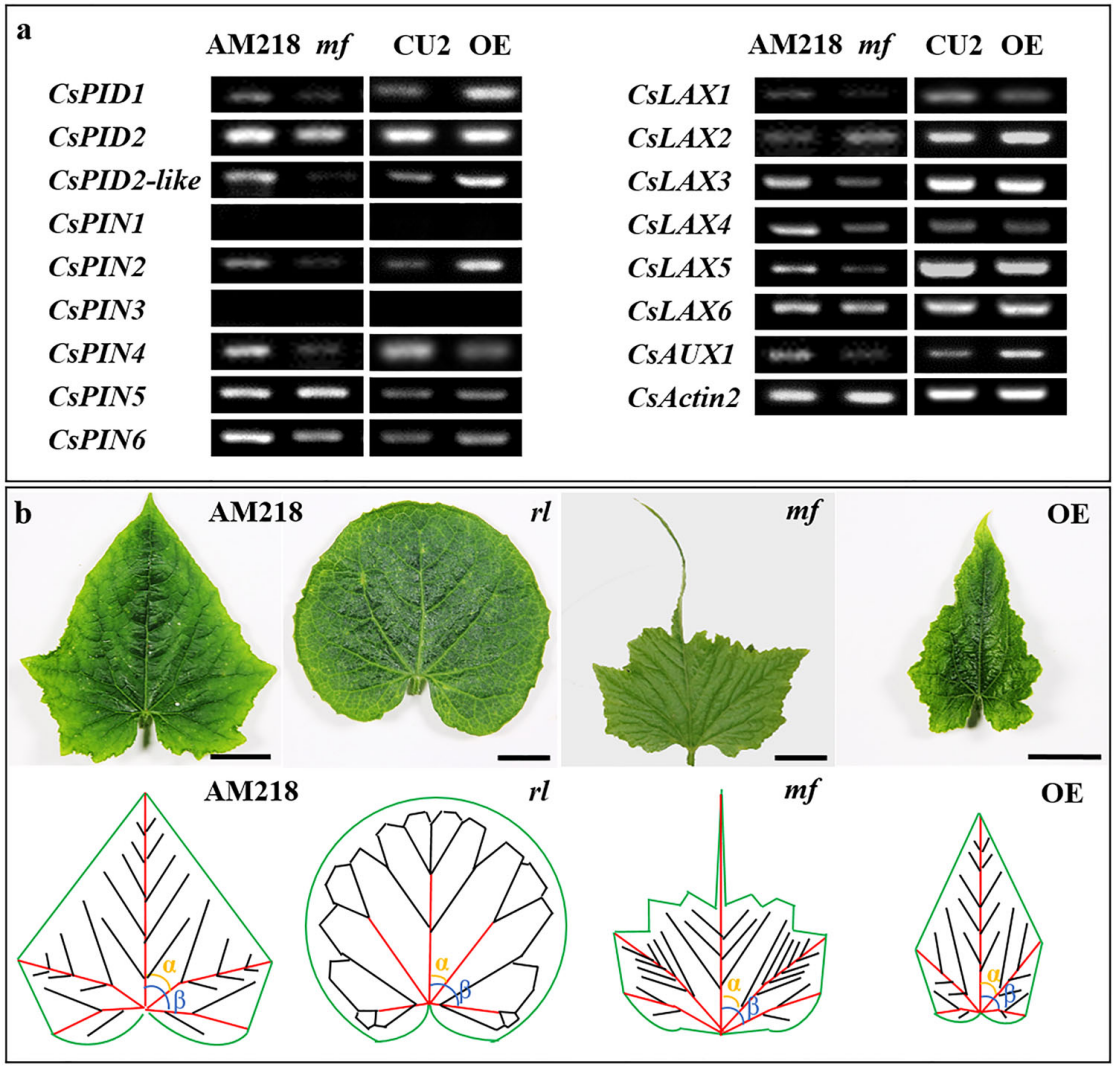
Comparison of auxin polar transport-related gene expression and leaf shapes in different plants. **a** Semiquantitative PCR assay of the genes involved in auxin polar transport in leaves of the *mf* mutant, *CsWOX1*-OE (OE), and their corresponding wild types (AM218 and CU2, respectively). **b** Leaf shapes at the 6th leaf position from the plant apex in the WT (AM218), *rl*, *mf*, and OE plants. A schematic illustration of the vein distribution is shown below each leaf. Primary veins extending from the petiole are shown in red; the black line represents the secondary veins. *α*, the angle between the midvein and adjacent primary vein; *β*, the angle between the midvein and separated primary vein. Bar = 5 mm. Six biological repeats were included per measurement

The T-DNA domain of the overexpression vector and upregulated expression of *CsWOX1* were detected in the leaves with vein defects in the other two plants (Supplemental Fig. S4). In L1 (Line 1) leaves, the two additional primary veins adjacent to the midvein extended to the two extra leaf tips. In L2 (Line 2) leaves, six primary veins extended to six leaf tips symmetrically.

### Genes associated with the auxin polar transport response to CsWOX1 function in leaf development

We then assessed whether loss or gain of CsWOX1 function is correlated with changes in the transcription of genes associated with auxin polar transport. The auxin polar transport mediators PIDs and the auxin efflux carriers PINs were reported previously^34^. In this study, auxin influx carriers (*AUX*/*LAX* family genes) were identified in the cucumber genome database (Supplemental Figs. S5 and S6; Supplemental Table S3). In the *mf* leaves, expression of *PID1* (mutant gene in the cucumber ‘*round leaf*’ line), *PID2-like*, three *PINs* (*PIN2*, *PIN4*, and *PIN6*), and five *LAXs* (*LAX1*, *LAX3*, *LAX4*, *LAX5*, and *AUX1*) were downregulated compared with that in the AM218 plants. In contrast, two *PIDs* (*PID1* and *PID2-like*), two *PINs* (*PIN2* and *PIN6*), and *AUX1* were upregulated in the *CsWOX1*-OE plants compared with the CU2 plants (Fig. 5a). Upregulation of these auxin polar transport-related genes in the *CsWOX1*-OE plants showed an opposite trend to the *mf* mutant plants, suggesting that CsWOX1 positively regulated the transcription of these genes.

### Leaf morphogenesis correlated with changes in vein pattern

To analyze the relationship between venation and leaf shape, we also examined the primary and secondary veins and margins in mature leaves at the sixth leaf position from the top of 1-month-old plants (AM218, *rl*, *mf*, and *CsWOX1-*OE). In Fig. 5b, primary veins extending from the petiole are shown in red, and the black lines represent the secondary veins. In the *mf* leaves, secondary veins with shorter lengths and higher densities than those in the WT leaves were found in the remaining proximal part of the leaf (Fig. 5b; Supplemental Table S4). In the *rl* leaves, primary veins with a decreased average length and secondary veins with an increased average length can be connected to each other to form loops (Fig. 5b; Supplemental Table S4). In the *CsWOX1-*OE leaves, there was a higher density of primary veins and a lower density of secondary veins than those of the WT leaves (Fig. 5b; Supplemental Table S4). In addition, smaller angles (*α* and *β* in Fig. 5b) between the primary veins were observed in the *mf* and *CsWOX1-*OE leaves than the WT leaves (Fig. 5b; Supplemental Table S4).

### CsWOX1 interacts with CsTCP4a

**Fig. 6:**
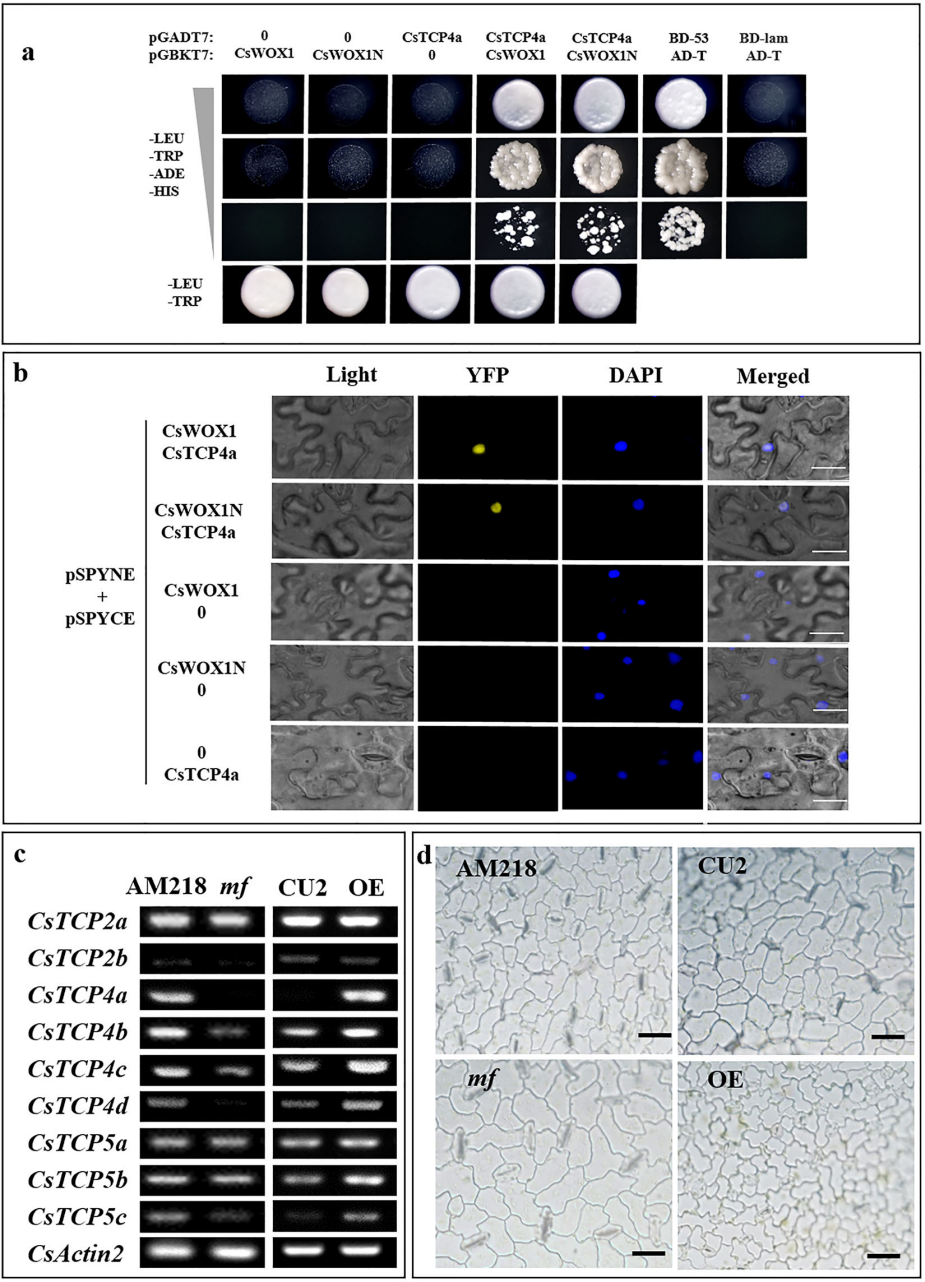
Interaction between CsWOX1 and TCP and the effects on the subepidermal cell size of the leaves. **a** Yeast two-hybrid assay showing direct interactions between both CsWOX1 and mutant Cswox1 (CsWOX1N) and CsTCP4a at the protein level. Vector combinations of pGBKT7-53/pGADT7-T and pGBKT7-lam/pGADT7-T were used as the positive and negative controls, respectively. A yeast concentration gradient (10^−1^, 10^−2^, 10^−3^) was tested for growth on selective media (-leu, -trp, -his, -ade) and compared to growth on nonselective media (-leu, -trp). **b** Physical interactions between CsWOX1/CsWOX1N and CsTCP4a were detected in the leaves of *Nicotiana benthamiana* using BiFC assays. **c** Expression patterns of TCP family genes in the *mf* mutant, *CsWOX1*-OE (OE), AM218, and CU2 lines. **d** Leaf cell sizes in the 3rd leaf from the top in all plants (AM218, *mf*, CU2, and *CsWOX1*-OE). Bar = 30 µm (**b**), 20 µm (**d**)

To identify putative proteins that interact with CsWOX1, we performed a yeast two-hybrid (Y2H) assay, and 30 proteins were found to putatively interact with CsWOX1. One of the putative proteins, Csa4G088720, predicted as a TCP transcription factor (CsTCP4a), was confirmed to interact by a further Y2H test (Fig. 6a). In addition, CsWOX1N, which lacks the conserved WUS box of the C-terminus, maintained a physical interaction with CsTCP4a, suggesting that the interaction with CsTCP4a is dependent on the N-terminus of CsWOX1. A bimolecular fluorescence complementation (BiFC) assay in leaves of *N. benthamiana* confirmed the interaction between CsWOX1 and CsTCP4a, as well as CsWOX1N and CsTCP4a (Fig. 6b). In addition, the expression of *CsTCP4a* was detected in leaf edge tissues at different stages (from 1 to 4 cm of leaf length) (Supplemental Fig. S7). CsTCP4a belongs to the CIN subfamily of TCPs. Because of the key role of CIN-TCP transcription factors in leaf development, homologous genes in cucumber were identified (Supplemental Fig. S8; Supplemental Table S3), and the expression of these genes was examined (Fig. 6c). The results showed that the expression of five *CIN-TCP* genes (*CsTCP4a*, *CsTCP4b*, *CsTCP4c*, *CsTCP4d*, and *CsTCP5c*) was lower in the *mf* leaves than in the AM218 leaves. Moreover, in the *CsWOX1*-OE leaves, the expression of six *CIN-TCPs* (*CsTCP4a*, *CsTCP4b*, *CsTCP4c*, *CsTCP4d*, *CsTCP5b*, and *CsTCP5c*) was higher than that in the CU2 leaves (Fig. 6c). These results indicate that the expression of these *CIN-TCP* genes tends to show a reverse trend between *CsWOX1*-OE and *mf*, suggesting that CsWOX1 may negatively regulate these *CIN-TCPs*.

We also observed the subepidermal cells of leaves from all cucumber plants examined (AM218, *mf*, CU2, and *CsWOX1-*OE). Leaves were sampled from the 3rd leaf position from the top. Compared with those of the AM218 plants, the lower epidermal cells from *mf* were significantly larger, and the ratio (*mf*/AM218) of cell area was ~1.89 (Fig. 6d; Supplemental Fig. S9). In contrast, the cell size of the *CsWOX1-*OE leaves decreased substantially compared with that of CU2 (Fig. 6d), and the ratio (*CsWOX1-*OE/CU2) of the cell area was ~0.70 (Supplemental Fig. S9). Similar to the gene expression pattern, this contrasting trend was also observed in cell expansion between *CsWOX1-*OE and *mf*.

## Discussion

### CsWOX1 is involved in cucumber vein development depending on auxin polar transport

In *Arabidopsis*, the *wox1 prs* double mutant showed a uniformly narrower leaf width from the base to the tip (Fig. 2a), while the distal part of the cucumber *mf* leaf was much narrower than the proximal part compared with AM218 (Fig. 1a). These differences in WOX1 homologous mutants may be related to the differing leaf vein structure of different species. In *P. x hybrida*, *M. truncatula*, *N. sylvestris*, *P. sativum* L., *Arabidopsis thaliana*, and *Z. mays* L., the leaves have only one primary vein extending from the petiole, and then, the secondary veins extend from the main vein to the leaf margin. However, in cucumber, the normal leaf vein pattern is palmate, with five primary veins extending from the petiole to the leaf margin (Figs. 3b and 5b). In the *mf* mutant, the middle vein grew normally, while defects in the length of the secondary vein and smaller angles (α and β) between the primary veins (Fig. 5b; Supplemental Table S4) occurred in the remaining proximal leaf tissue. Considering that all the WOX1 homologous mutants exhibited a normal leaf length, the mutant phenotype of the mediolateral axis in mf leaf veins, as well as other species, suggests that WOX1 may participate in the formation of the leaf vein pattern.

We then compared the vein pattern in Fig. 5b and revealed that the length, number, density and angle of the leaf veins are all linked with the shape of the cucumber leaves studied (Supplemental Table S4). In the *CsWOX1*-OE leaves, narrow blades have smaller angles between the primary veins, and the ‘butterfly-shaped’ leaves with seven primary veins extend from the petiole produced (Fig. 4f). In Supplemental Fig. S4a, similar changes in leaf vein pattern were observed with a corresponding increase in primary veins and leaf tips in the other *CsWOX1*-OE leaves. In the *mf* leaves, the loss of distal tissue and margin corresponds to a shorter length of the secondary veins and smaller angles (*α* and *β*) between the primary veins. In the *rl* leaves, round leaves indicated that primary veins with large angle *β* bifurcate before reaching the leaf margin and form loops with the secondary and minor veins. These findings agree with the hypothesis that changes in leaf shape and size are correlated with changes in leaf vein pattern and density^35,36^. Therefore, CsWOX1 can participate in the formation of leaf shapes with normal vein patterns.

Semiquantitative PCR revealed downregulation of two *PIDs*, three *PINs*, and five *LAXs* in the *mf* plants and significant upregulation of two *PIDs*, two *PINs*, and two *LAXs* in the *CsWOX1*-OE plants (Fig. 5a). These findings suggest that CsWOX1 positively regulates the expression of auxin polar transport carriers. In *Arabidopsis*, *PINs* play roles in the intracellular auxin-transport pathway and the formation of vein patterning^37^. Moreover, the auxin influx carrier *LAX2* is known to control vasculature development^14^. In the *mf* leaves, semiquantitative detection showed that the expression levels of *PINs* and *PIDs* decreased (Fig. 5a), which can affect the level of auxin polar transport^37,38^. In the *rl* leaves, CsPINs with abnormal polarity localization due to the loss of phosphorylation function of CsPID1 may result in dysfunction of auxin polar transport^16,33,38,39^. Therefore, the *mf rl* double mutant showed a similar leaf and petal shape to the *rl* mutant (*Cspid* mutant), indicating that CsWOX1 may be involved in the formation of leaf morphology depending on auxin transport, which is most likely mediated by CsPID1.

In *Arabidopsis*, it has been revealed that auxin signaling plays a regulatory role upstream of WOX1 during leaf development, limiting the expression of *WOX1* in leaf margins^3,9^. In this study, we found that CsWOX1 can participate in the formation of leaf vein patterns depending on auxin polar transport-related genes (Fig. 5a; Supplemental Table S4). This finding complements the interaction relationship between WOX1 and auxin during leaf development. Therefore, we found that WOX1 not only acts as a receptor sensing the limitation from auxin signaling but also as a regulator that participates in auxin polar transport in the leaf restricted region.

### CsWOX1 regulates leaf size via the CIN-TCP transcription factors

In species with simple leaves, mutations of CIN-TCPs result in changes in leaf size, shape and curvature, consistent with their roles as growth repressors^40^. Five miR319-targeted CIN-TCPs in *Arabidopsis*, TCP2, 3, 4, 10, and 24, and their orthologs in snapdragon and tomato, were found to control leaf morphogenesis by limiting the number of pavement cells^22,25,41,42^. CsTCP4a was stably expressed in the marginal tissue at different leaf development stages (Supplemental Fig. S7). CsWOX1 was found to physically interact with CsTCP4a, and the interaction with CsTCP4a is dependent on the N-terminus of CsWOX1 (Fig. 6a, b). Therefore, CsWOX1 may be involved in the regulation of cell division during leaf development through a protein complex formed with CsTCP4a. In *mf*, structural variation of the protein complex due to the CsWOX1 mutation may influence the role of CsTCP4a in the duration of cell division. In the *mf* and *CsWOX1*-OE leaves, five *CIN-TCPs* and six *CIN-TCPs* were down- and upregulated, respectively (Fig. 6c). These results suggesting that CsWOX1 regulates the *CIN-TCP* genes at the protein and transcriptional levels. Consistent with the phenotypes of leaves with elevated activity of CIN-TCP proteins in *Arabidopsis*^41,43^, the cucumber *CsWOX1*-OE leaves with upregulated *CIN-TCP* genes were smaller in size. *CsWOX1* is therefore thought to affect leaf expansion via cell proliferation dependent on the *CIN-TCP* genes. In this study, the decrease in leaf size in *CsWOX1-*OE compared with CU2 is thought to be dependent on the upregulation of the *CIN-TCP* genes (Fig. 6c). This finding can complement the regulatory mechanism between WOXs and TCPs during leaf development.

The proportion (*CsWOX1*-OE/CU2) of leaf area in the 1st to 4th leaves was ~0.6 and that in the 5th and 6th leaves was ~0.45 (Fig. 4a, e). Leaf size mainly relies on both cell proliferation and cell expansion to promote leaf growth^44–46^. The cell number and size of the lower epidermis of the *CsWOX1*-OE and *mf* leaves under the same conditions of microscopic observation showed an opposite trend separately compared with the wild type (CU2/AM218) (Fig. 6). CIN-TCPs are negative regulators of the duration of the proliferation phase and control the transition from cell proliferation to expansion during leaf morphogenesis in dicotyledons^40,44^. Therefore, CsWOX1 may affect the cell developmental process depending on the positive regulation and protein interaction with CIN-TCPs, which can change the final leaf size.

In summary, this study revealed the function of CsWOX1 in leaf vein pattern and leaf size. CsWOX1 can participate in leaf vein formation, and leaf vein patterns depend on the regulation of auxin polar transport-related genes. Regarding leaf size, CsWOX1 may participate in the process of cell proliferation depending on the protein interaction between CsWOX1 and CsTCP4a and the expression regulation of *CIN-TCPs* by CsWOX1. These results provide support for the further analysis of WOX1’s function in leaf development. However, the downstream regulators of CsWOX1, such as the regulators involved in cell expansion, need to be further investigated in leaf development.

## Materials and methods

### Plant material development and phenotypic characterization

In this study, the cucumber mutant line *mf*, which represents a spontaneous mutation of the *CsWOX1* gene in the cucumber inbred line ‘Extra Early Majestic’ (AM218), was used to analyze leaf development. Map-based cloning has shown that the *mf* mutant encodes a truncated WOX1 transcription factor, which was responsible for the mutant phenotype in *mf* mutants^28^. All populations were grown in the greenhouses of Northwest A&F University, Yangling, China. *Arabidopsis* ecotype Columbia (Col-0) was used for transgenic investigation. The cucumber line *round leaf* (*rl*) with a mutant *CsPID1* gene was identified in an EMS-mutagenized population^33^. All *Arabidopsis* plants and cucumber seedlings were grown in growth chambers under 16 h light at 22 °C/8 h dark at 18 °C and 16 h light at 28 °C/8 h dark at 18 °C, respectively.

Microscopic phenotyping of *Arabidopsis* flowers was performed by a stereomicroscope (S8APO, Leica, Germany). One-month-old Col-0, *Atwox1 prs*, and *35S:CsWOX1 Atwox1 prs* plants were photographed. The width of the sixth rosette leaf was measured when it was fully expanded. Flowers in the top inflorescence of the main stem in one-and-a-half-month-old plants were selected, and their petals were photographed under a stereomicroscope (S8APO, Leica) and then used to measure the width. Ten biological repeats were included per measurement.

Young cucumber leaves from 1-month-old AM218 and *mf* plants were used for scanning electron microscopy (SEM) assays. The leaves were fixed, dried, dissected, coated according to a previous description^47^, and then observed under a scanning electron microscope (JSM-6360, JEOL, Japan).

### Genome-wide identification and phylogenetic analysis of AUX/LAX family genes in the cucumber genome

Sequences of *AUX*/*LAX* family genes in *Arabidopsis* were obtained from TAIR (https://www.arabidopsis.org/) and then blasted into the cucumber genome database (CuGenDB, http://cucurbitgenomics.org/) to reveal the homologous genes. All genes from *Arabidopsis* and cucumber were aligned using Clustal W2 (http://www.ebi.ac.uk/Tools/msa/clustalw2/). Then, an unrooted neighbor-joining (NJ) phylogenetic tree was constructed using MEGA 5.10 software with 1000 bootstrap replications, pairwise deletion and a Poisson model. All gene-specific primers are listed in Supplemental Table S5.

### Semiquantitative reverse transcription PCR analysis

Young leaves (~1 cm) from 1-month-old AM218 plants, *mf* mutant plants, CU2, plants overexpressing *CsWOX1* (*CsWOX1*-OE), *rl* mutant plants, and *mf rl* double mutant plants were collected for RNA extraction. Total RNA isolation, first-strand cDNA synthesis, and reverse transcription (RT) PCR were performed as described previously^48^. *CsActin2* was used to normalize the expression data^49^. Three biological and three technical repeats were carried out for each gene. All gene-specific primers are listed in Supplemental Table S5.

### Transcriptome profiling of the WT (AM218) and *mf* plants using RNA-Seq

Young leaves (~1 cm) from 1-month-old AM218 and *mf* plants were used for RNA-Seq analysis. Leaves from the same plant were pooled as one biological replicate, and three biological repeats were used per genotype. RNA-Seq libraries were constructed according to a previous description^28^. Genes with at least a 2-fold change in expression between the AM218 and *mf* plants and a false discovery rate (FDR) of <0.05 were considered differentially expressed. The cucumber ‘Chinese Long’ genome (http://cucurbitgenomics.org/organism/2) was used as the reference genome.

### RNA in situ hybridization

The apical buds containing young leaves and leaf primordia were fixed in 3.7% formalin–acetic acid–alcohol (FAA) solution and used for in situ hybridization (ISH) as described previously^50^. Sense and antisense probes were synthesized using SP6 and T7 polymerase, respectively. Probes for *CsWOX1* were designed using the 5′-region of the coding sequence (CDS). Primers for probe generation are listed in Supplemental Table S5.

### Comparison of leaf size and shape, leaf epidermal cell area, and leaf vein characteristics in cucumber leaves

Cucumber leaves were collected from the same position on same-age seedlings of the AM218, CU2, *mf* mutant, and *CsWOX1*-OE lines. Then, the size of the leaves and their subepidermal cells were measured. After photos were taken of the leaves at the same blade position, image analysis software (Cell Standard, Olympus, Japan) was used to measure the relative blade length and blade area in pixel and pixel^2^ units, respectively. The average width was then calculated from the ratio of blade area to length followed by the ratio of the average width to length. Five biological repeats were included per measurement.

Cell area was measured in the 3rd leaf from the top in same-age seedlings. Then, the subepidermal cells were peeled off gently from the same leaf using tweezers. Pictures of the cells were taken under ×10 (eyepiece) and ×40 (objective) magnification using a microscope (BX63, Olympus). Ten cells in each view were then randomly selected for measurements of the relative area in pixel^2^ units using Cell Standard (Olympus). Three biological repeats were included per measurement.

Leaf veins were classified according to branching architecture^51^. The primary veins connect directly to the petiole, the secondary veins branch from the primary veins, and the tertiary veins are smaller in diameter, branching from and sometimes linking the primary and secondary veins. The higher-order “minor veins” with smaller diameters form a continuous mesh with the major veins^51^. Mature leaves at the sixth leaf position from the plant apex were collected from 1-month-old plants of the WT (AM218), *mf* mutant, *rl* mutant, and *CsWOX1*-OE lines. The leaf area and length of the primary and secondary leaf veins were measured by the measurement software ImageJ (National Institutes of Health, America). A protractor was used to measure angle *α* (the angle between the midvein and adjacent primary vein) and angle *β* (the angle between the midvein and separated primary vein). Six biological repeats were included per measurement.

### Yeast two-hybrid and bimolecular fluorescence complementation assays

The yeast two-hybrid (Y2H) assay was conducted as described previously^28^. Mixed samples of apical buds and young leaves (~1 cm) from cucumber ‘Chinese Long’ were taken to construct a yeast two-hybrid library. The full-length CDS of *CsTCP4a* was cloned into pGADT7 bait, and those of *CsWOX1* and *Cswox1* (the mutant coding sequence of *CsWOX1*) were cloned into pGBKT7 prey vectors. All recombinant constructs were verified by sequencing and then transformed into the yeast strain AH109. The combination of pGBKT7-53 and pGADT7-T was used as the positive control, with the combination of pGBKT7-lam and pGADT7-T as the negative control (Clontech, China). For the bimolecular fluorescence complementation (BiFC) assay, full-length CDSs of *CsWOX1*, *Cswox1*, and *CsTCP4a* without stop codons were cloned into pSPYNE-35S and pSPYCE-35S vectors containing each half of the yellow fluorescent protein (YFP) to recombine the fusion proteins. Recombinant plasmids were transformed into *Agrobacterium tumefaciens* strain GV3101 and then cotransformed into the abaxial sides of 4- to 6-week-old *N. benthamiana* leaves using sterile syringes^52^. The leaves of *N. benthamiana* were visualized using a fluorescence microscope (BX63, Olympus) within 48–72 h.

### CRISPR-Cas9 targeting of *AtWOX1* and *PRS* (*AtWOX3*) genes and ectopic expression of *CsWOX1* in *Arabidopsis*

For CRISPR-Cas9 targeting assays, specific candidate sites of *AtWOX1* and *AtWOX3* were predicted. The target sequences were synthesized and fused with the BsaI-linearized CRISPR-Cas9 binary vector pHEE40E1. The construct was then transformed into *A. tumefaciens* strain GV3101 and used for *Agrobacterium*-mediated transformation in *Arabidopsis*^53^. Plants including T-DNA were screened on Murashige and Skoog medium with 50 mg L^−1^ hygromycin (Sigma, China) and identified using PCR. Positive knockout lines (*wox1 prs* double mutants) were verified by sequencing the target sequence and phenotyping as described previously^5^.

The full-length CDS of the *CsWOX1* gene was amplified and cloned into the pBI121 vector^28^. The recombinant plasmids were introduced into *A. tumefaciens* strain GV3101 and transformed into the *wox1 prs* double mutant. Primers used in gene knockout and overexpression are listed in Supplemental Table S5.

### Transformation of cucumber and fluorescence detection of transgenic cucumber roots

The full-length CDS of *CsWOX1* was inserted into the pCAMBIA1305.4 vector with GFP protein under the control of the 35S promoter and then transformed into *A. tumefaciens* strain EHA105. Bacteria containing the construct were then transferred into the cucumber inbred line CU2 as described previously^54^ and generated the *CsWOX1* overexpression line (*CsWOX1*-OE).

Roots of the *CsWOX1*-OE and CU2 plants were collected in the greenhouse. Green fluorescence was observed by a stereoscopic fluorescence microscope (MZ10F, Leica). Pictures were taken in GFP and bright channels, with the corresponding rulers^28^.

## Supplementary information

Supplemental Figures-revised

Supplemental Tables-revised

Supplemental Figures

Supplemental Tables

## Data Availability

The data that support the results are included in this article and its supplementary materials. Other relevant materials are available from the corresponding author upon reasonable request.

## References

[1] Timmermans MC, Schultes NP, Jankovsky JP, Nelson T (1998). *Leafbladeless1* is required for dorsoventrality of lateral organs in maize. Development.

[2] Hagemann W, Gleissberg S (1996). Organogenetic capacity of leaves: the significance of marginal blastozones in angiosperms. Plant Syst. Evol..

[3] Guan C (2017). Spatial auxin signaling controls leaf flattening in *Arabidopsis*. Curr. Biol..

[4] Vandenbussche M (2009). Differential recruitment of *WOX* transcription factors for lateral development and organ fusion in Petunia and *Arabidopsis*. Plant Cell.

[5] Nakata M (2012). Roles of the middle domain-specific *WUSCHEL-RELATED HOMEOBOX* genes in early development of leaves in *Arabidopsis*. Plant Cell.

[6] Nardmann J, Ji JB, Werr W, Scanlon MJ (2004). The maize duplicate genes *narrow sheath1* and *narrow sheath2* encode a conserved homeobox gene function in a lateral domain of shoot apical meristems. Development.

[7] Tadege M (2011). STENOFOLIA regulates blade outgrowth and leaf vascular patterning in *Medicago truncatula* and *Nicotiana sylvestris*. Plant Cell.

[8] Zhuang LL (2012). LATHYROIDES, encoding a WUSCHEL-related Homeobox1 transcription factor, controls organ lateral growth, and regulates tendril and dorsal petal identities in garden pea (*Pisum sativum* L.). Mol. Plant.

[9] Du F, Guan C, Jiao Y (2018). Molecular mechanisms of leaf morphogenesis. Mol. Plant.

[10] Pekker I, Alvarez JP, Eshed Y (2005). Auxin response factors mediate *Arabidopsis* organ asymmetry via modulation of KANADI activity. Plant Cell.

[11] Blakeslee J, Peer W, Murphy A (2005). Auxin transport. Curr. Opin. Plant Biol..

[12] Galweiler L (1998). Regulation of polar auxin transport by AtPIN1 in *Arabidopsis* vascular tissue. Science.

[13] Mattsson J, Sung ZR, Berleth T (1999). Responses of plant vascular systems to auxin transport inhibition. Development.

[14] Moreno JE, Romani F, Chan RL (2018). *Arabidopsis thaliana* homeodomain-leucine zipper type I transcription factors contribute to control leaf venation patterning. Plant Signal Behav..

[15] Fabregas N (2015). Auxin influx carriers control vascular patterning and xylem differentiation in *Arabidopsis thaliana*. PLoS Genet..

[16] Benjamins R, Quint A, Weijers D, Hooykaas P, Offringa R (2001). The PINOID protein kinase regulates organ development in *Arabidopsis* by enhancing polar auxin transport. Development.

[17] Christensen S, Dagenais N, Chory J, Weigel D (2000). Regulation of auxin response by the protein kinase PINOID. Cell.

[18] Morita Y, Kyozuka J (2007). Characterization of OsPID, the rice ortholog of PINOID, and its possible involvement in the control of polar auxin transport. Plant Cell Physiol..

[19] Bai F (2005). Molecular characterization and expression of *PsPK2*, a *PINOID-like* gene from pea (*Pisum sativum*). Plant Sci..

[20] Nakata M, Okada K (2012). The three-domain model: a new model for the early development of leaves in *Arabidopsis thaliana*. Plant Signal Behav..

[21] Zhang F (2014). STENOFOLIA recruits TOPLESS to repress *ASYMMETRIC LEAVES2* at the leaf margin and promote leaf blade outgrowth in *Medicago truncatula*. Plant Cell.

[22] Ori N (2007). Regulation of *LANCEOLATE* by miR319 is required for compound-leaf development in tomato. Nat. Genet..

[23] Alvarez JP, Furumizu C, Efroni I, Eshed Y, Bowman JL (2016). Active suppression of a leaf meristem orchestrates determinate leaf growth. eLife.

[24] Bresso EG, Chorostecki U, Rodriguez RE, Palatnik JF, Schommer C (2017). Spatial control of gene expression by miR319-regulated TCP transcription factors in leaf development. Plant Physiol..

[25] Palatnik JF (2003). Control of leaf morphogenesis by microRNAs. Nature.

[26] Efroni I, Blum E, Goldshmidt A, Eshed Y (2008). A protracted and dynamic maturation schedule underlies *Arabidopsis* leaf development. Plant Cell.

[27] Sack L, Dietrich EM, Streeter CM, Sánchez-Gómez D, Holbrook NM (2008). Leaf palmate venation and vascular redundancy confer tolerance of hydraulic disruption. Proc. Natl Acad. Sci. USA.

[28] Niu H (2018). The *WUSCHEL-related homeobox1* gene of cucumber regulates reproductive organ development. J. Exp. Bot..

[29] Matsumoto N, Okada K (2001). A homeobox gene, *PRESSED FLOWER*, regulates lateral axi-dependent development of *Arabidopsis* flowers. Genes Dev..

[30] Xi W, Gong X, Yang Q, Yu H, Liou YC (2016). Pin1At regulates PIN1 polar localization and root gravitropism. Nat. Commun..

[31] Verrier PJ (2008). Plant ABC proteins—a unified nomenclature and updated inventory. Trends Plant Sci..

[32] Titapiwatanakun B (2009). ABCB19/PGP19 stabilises PIN1 in membrane microdomains in *Arabidopsis*. Plant J..

[33] Liu X (2019). PINOID is required for lateral organ morphogenesis and ovule development in cucumber. J. Exp. Bot..

[34] Zhang C (2018). Mutations in *CsPID* encoding a Ser/Thr protein kinase are responsible for round leaf shape in cucumber (*Cucumis sativus* L.). Theor. Appl. Genet..

[35] Dengler N, Kang J (2001). Vascular patterning and leaf shape. Curr. Opin. Plant Bio..

[36] Pahari S (2014). *Arabidopsis* UNHINGED encodes a VPS51 homolog and reveals a role for the GARP complex in leaf shape and vein patterning. Development.

[37] Sawchuk MG, Scarpella E (2013). Control of vein patterning by intracellular auxin transport. Plant Signal Behav..

[38] Friml J (2004). A PINOID-dependent binary switch in apical-basal PIN polar targeting directs auxin efflux. Science.

[39] Pressoir G (2009). Natural variation in maize architecture is mediated by allelic differences at the PINOID co-ortholog *barren inflorescence2*. Plant J..

[40] Sarvepalli K, Nath U (2018). CIN-TCP transcription factors: transiting cell proliferation in plants. IUBMB Life.

[41] Schommer C (2014). Repression of cell proliferation by miR319-regulated TCP4. Mol. Plant.

[42] Karidas P, Challa KR, Nath U (2015). The *tarani* mutation alters surface curvature in *Arabidopsis* leaves by perturbing the patterns of surface expansion and cell division. J. Exp. Bot..

[43] Challa KR, Rath M, Nath U (2019). The CIN-TCP transcription factors promote commitment to differentiation in *Arabidopsis* leaf pavement cells via both auxin-dependent and independent pathways. PLoS Genet..

[44] Utpal N, Crawford BCW, Rosemary C, Enrico C (2003). Genetic control of surface curvature. Science.

[45] Sablowski R, Carnier DM (2014). Interplay between cell growth and cell cycle in plants. J. Exp. Bot..

[46] Czesnick H, Lenhard M (2015). Size control in plants–lessons from leaves and flowers. Cold Spring Harb. Perspect. Biol..

[47] Bai SL (2004). Developmental analyses reveal early arrests of the spore-bearing parts of reproductive organs in unisexual flowers of cucumber (*Cucumis sativus* L.). Planta.

[48] Li Z (2012). A putative positive feedback regulation mechanism in *CsACS2* expression suggests a modified model for sex determination in cucumber (*Cucumis sativus* L.). J. Exp. Bot..

[49] Wang H (2014). Identification of two cucumber putative silicon transporter genes in *Cucumis sativu*s. J. Plant Growth Regul..

[50] Zhang X (2013). Transcription repressor HANABA TARANU controls flower development by integrating the actions of multiple hormones, floral organ specification genes, and GATA3 family genes in *Arabidopsis*. Plant Cell.

[51] Nelson T, Dengler N (1997). Leaf vascular pattern formation. Plant Cell.

[52] Tao Q (2018). Ethylene responsive factor ERF110 mediates ethylene-regulated transcription of a sex determination-related orthologous gene in two *Cucumis species*. J. Exp. Bot..

[53] Clough SJ, Bent AF (1998). Floral dip: a simplified method for Agrobacterium-mediated transformation of *Arabidopsis thaliana*. Plant J..

[54] Hu B, Li D, Liu X, Qi J, Yang L (2017). Engineering non-transgenic gynoecious cucumber using an improved transformation protocol and optimized crispr/cas9 system. Mol. Plant.

