# Supplementary material for: WUSCHEL-related homeobox1 (WOX1) regulates vein patterning and leaf size in Cucumis sativus
Source: Hortic Res. 2020 Nov 1;7:182. doi: 10.1038/s41438-020-00404-y (PMC7603520; doi:10.1038/s41438-020-00404-y)
Supplement: Supplementary file 1 — Supplemental Figures-revised [file 41438_2020_404_MOESM1_ESM.pdf]

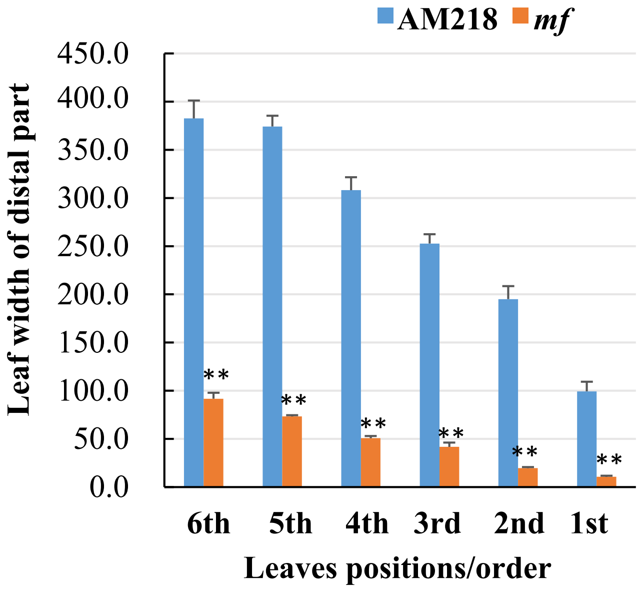

Supplemental Figure S1 The relative average width of the distal region in mf and AM218 leaves. Leaf distal region is the blade tissue from midpoint of midvein to leaf tip. Significance tests are performed using the Student's t-test method at 0.05 and 0.01 levels (*, p-value < 0.05; **, p-value < 0.01).

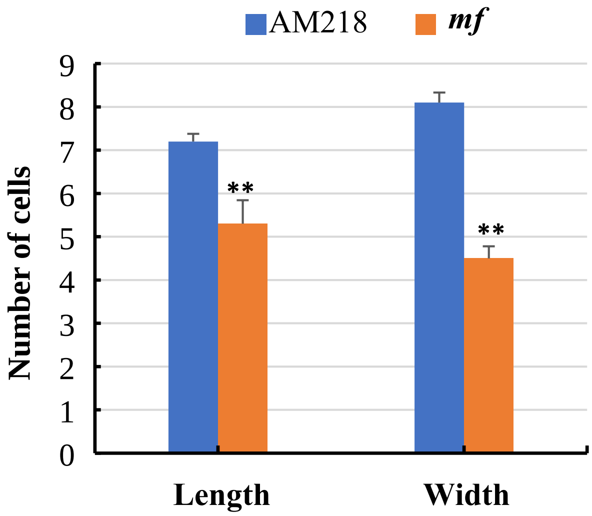

Supplemental Figure S2. Measurements of cell number per 200 µm × 200 µm leaf area in the AM218 wild-type and *mf* mutant. Values represent the mean and SE of 10 measurements from a SEM assay, and asterisks indicate a significant difference (*t* test, P < 0.01).

a

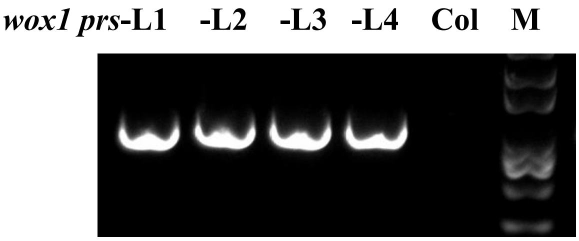

b

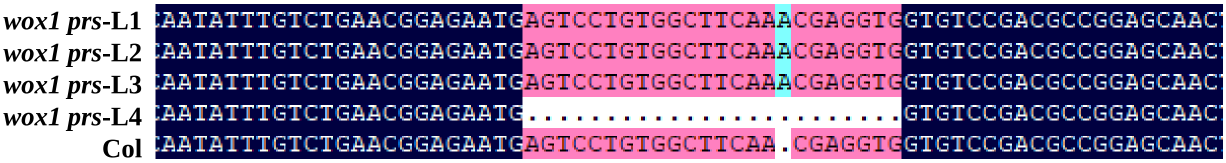

Target sequences of *WOX3* in *wox1 prs* lines

c

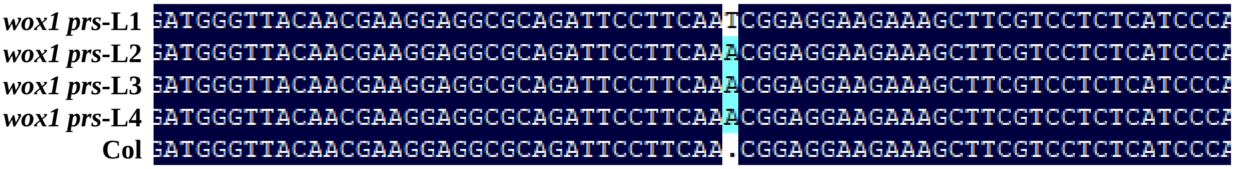

Target sequences of *WOX1* in *wox1 prs* lines

Supplemental Figure S3. Identification of *wox1 prs* double mutants in Arabidopsis. Four positive knockout lines (L1 to L4) were obtained from a CRISPR-Cas9 assay. a, PCR assay of the positive transgenic lines. M: DNA marker. b, Alignment of the target sequences of *WOX3* in the *wox1 prs* lines. c, Alignment of the target sequences of *WOX1* in the *wox1 prs* lines.

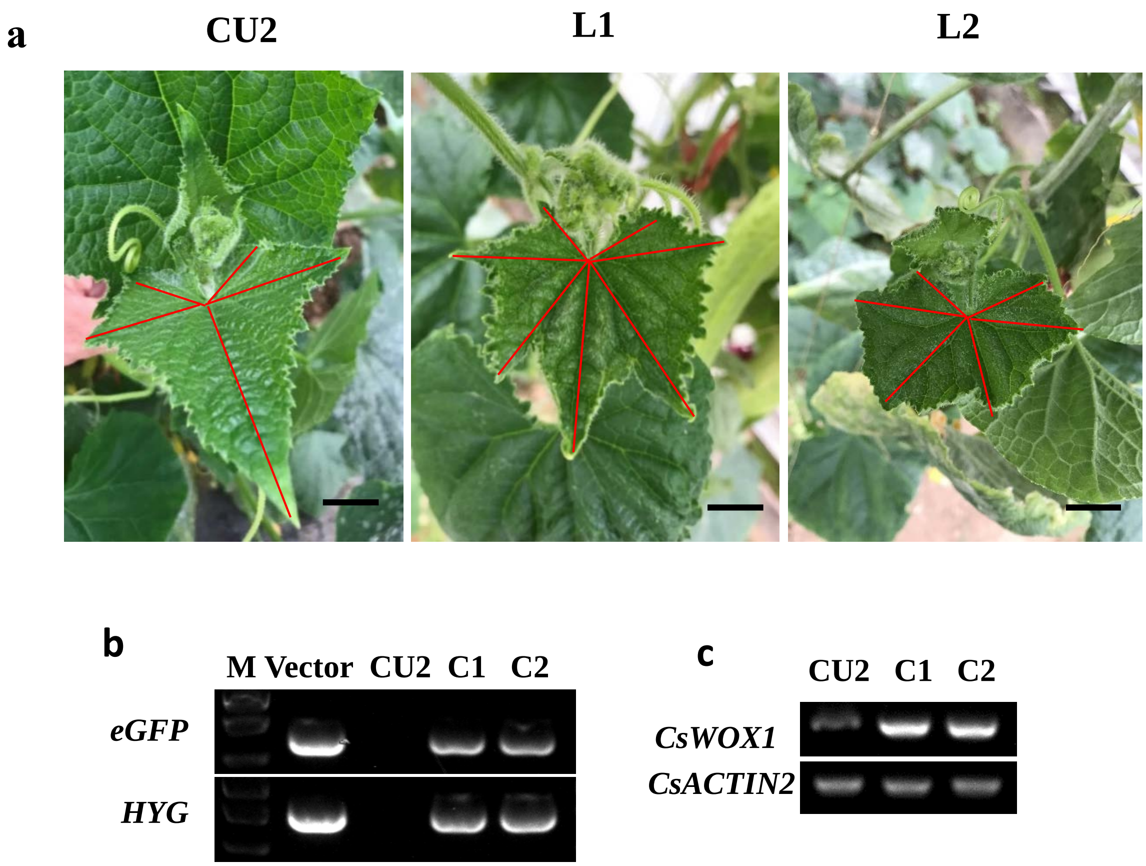

Supplemental Figure S4 The vein pattern variation of *CsWOX1*-OE leaves. a, Leaves in two lines of CsWOX1-OE and CU2. b, PCR assay in leaves of CsWOX1-OE lines and CU2. PCR targeted the CDS of the *eGFP* and *HYP* genes in the plasmid. M: DNA marker; L1: Line 1; L2: Line 2. c, Semi-quantitative PCR assay of the OE and CU2 leaves. The red lines represents the primary veins. Bar = 1 cm

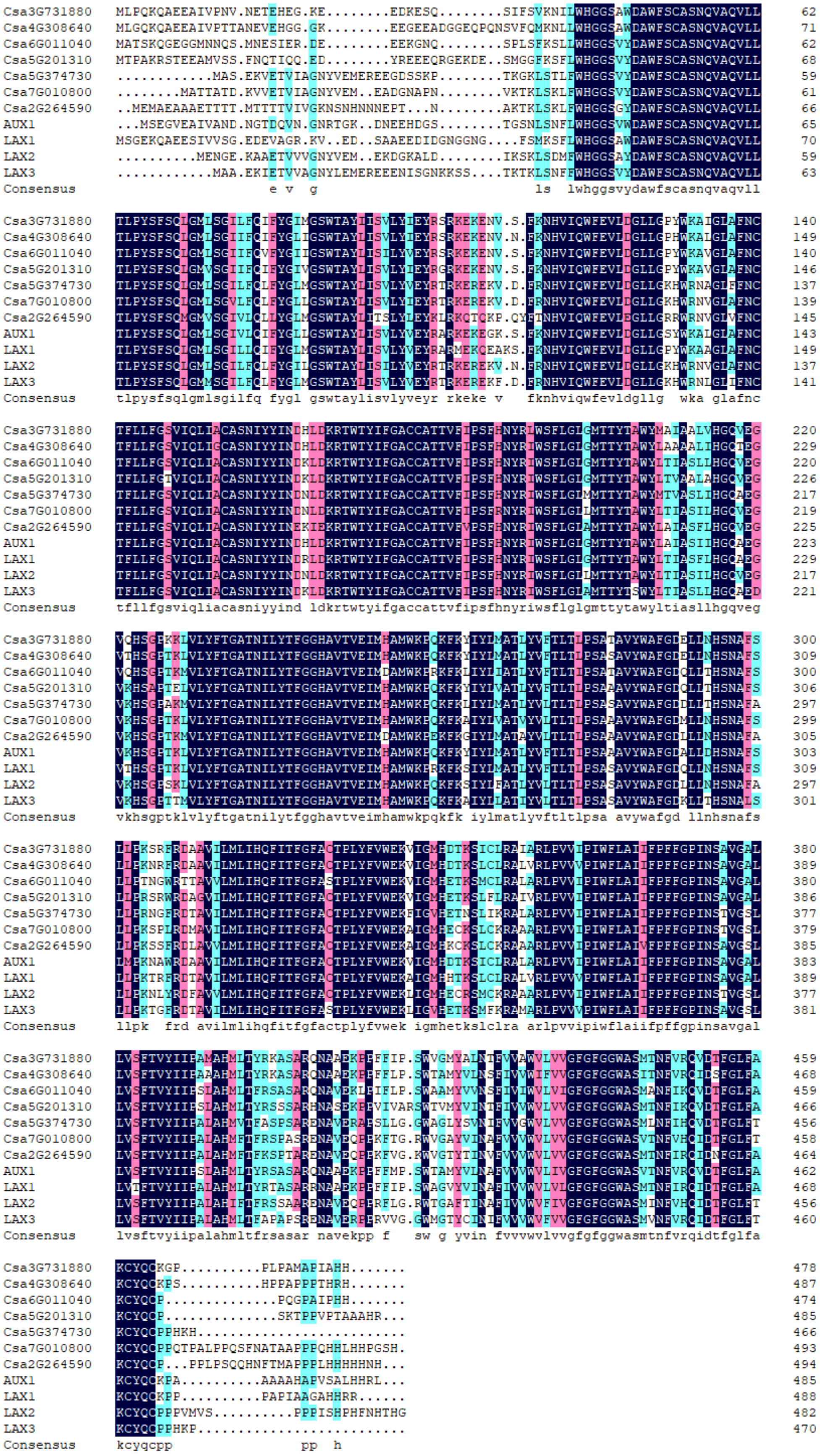

Supplemental Figure S5. Alignment of the amino acid sequences of seven AUX/LAX proteins in cucumber and four studied homologues in Arabidopsis. The gene names are listed in Supplemental Fig. S6 and Supplemental Table S3.

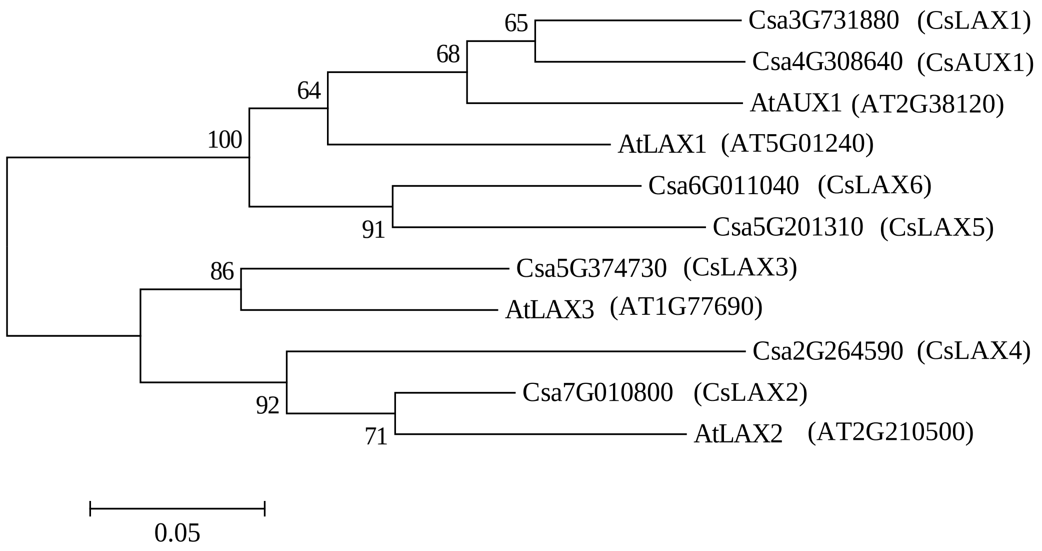

Supplemental Figure S6. Phylogram of the AUX/LAX family proteins based on the amino acid sequences of the four AtAUX/LAXs and seven CsAUX/LAXs shown in Supplemental Fig. S5.

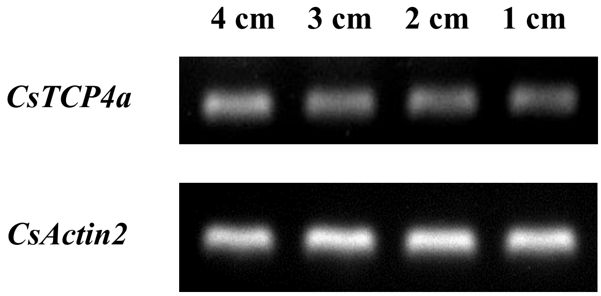

Supplemental Figure S7. The expression of *CsTCP4a* in cucumber leaf margin from different period. Leaf length is from 1 cm to 4 cm and the leaf margin is collected to extract RNA.

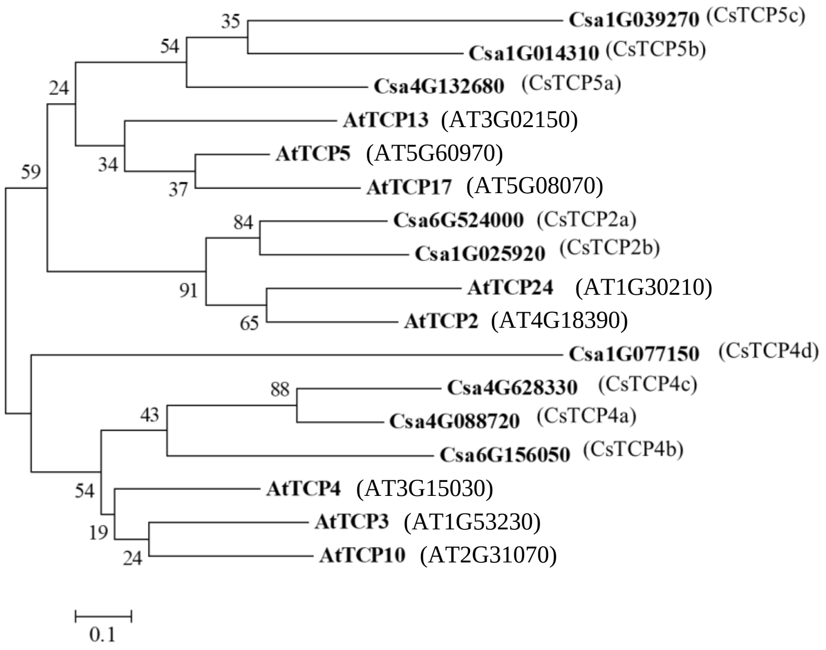

Supplemental Figure S8. Phylogram of the TCP family proteins based on the amino acid sequences from eight AtTCPs and nine CsTCPs. The gene names are listed in Supplemental Table S3.

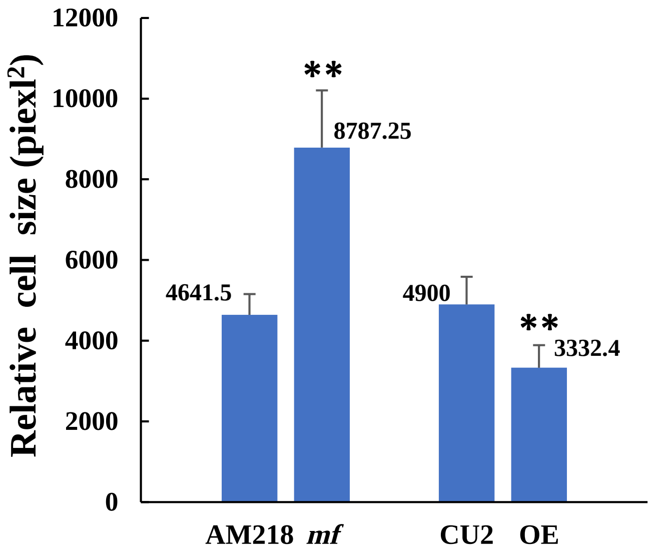

Supplemental Figure S9. Relative sizes of leaf sub-epidermal cells in different plants. Values represent the mean and SE of 10 measurements, and asterisks indicate a significant difference (*t* test, $P < 0.01$).
