# Supplementary material for: WUSCHEL-related homeobox1 (WOX1) regulates vein patterning and leaf size in Cucumis sativus
Source: Hortic Res. 2020 Nov 1;7:182. doi: 10.1038/s41438-020-00404-y (PMC7603520; doi:10.1038/s41438-020-00404-y)
Supplement: Supplementary file 2 — Supplemental Tables-revised [file 41438_2020_404_MOESM2_ESM.pdf]

**Supplemental Table S1. RNA-Seq analysis of differentially expressed genes in leaves of the *mf* mutant compared with its wild-type**

| No. | Gene ID | FDR | $log_2$FC | regulat | GO_annotation | KEGG_pathway_an | KOG_class_annotation | Pfam_annotation | Swiss-Prot_annotation | eggNOG_class_annotation | NR_annotation |
|---|---|---|---|---|---|---|---|---|---|---|---|
| 1 | Csa1G025050 | 1.88E-05 | 1.627441 | up | Biological Process: maltose metabolic process (GO:00000: | Glycerophospholipid | Lipid transport and metabolism;; Gener | Methyltransferase domain;; Methyltransferase domain;; Methyltran | Phosphoethanolamine n-methyltransfe | Lipid transport and metabolism | PREDICTED: phosphoethanolamine N-methyltransferase 1-like [Cucumis sativus] |
| 2 | Csa1G029620 | 3.15E-09 | 1.201244 | up | Molecular Function: binding (GO:0005488);; | -- | General function prediction only | Zinc finger, C2H2 type | Predicted protein GN=POPTRDRAF | General function prediction only | PREDICTED: uncharacterized protein LOC101209426 [Cucumis sativus] |
| 3 | Csa1G032440 | 0.026564 | -2.33846 | down | -- | -- | Posttranslational modification, protein t | Myb-like DNA-binding domain;; Myb-like DNA-binding domain | Predicted protein GN=POPTRDRAF | Transcription | PREDICTED: uncharacterized LOC101217341 [Cucumis sativus] |
| 4 | Csa1G032450 | 0.002226 | 1.422425 | up | Molecular Function: nucleic acid binding (GO:0003676);; N | -- | Transcription | BTB/POZ domain;; TAZ zinc finger | Protein binding protein, putative GN= | Transcription | PREDICTED: BTB/POZ and TAZ domain-containing protein 1-like [Cucumis sativus] |
| 5 | Csa1G042740 | 0.000913 | 1.233115 | up | Biological Process: protein peptidyl-prolyl isomerization (( | -- | Posttranslational modification, protein t | FKBP-type peptidyl-prolyl cis-trans isomerase | Peptidyl-prolyl cis-trans isomerase G | Posttranslational modification, protein turn | PREDICTED: peptidyl-prolyl cis-trans isomerase FKBP17-2, chloroplastic-like [Cucumis sativus] |
| 6 | Csa1G042780 | 1.73E-12 | 1.411164 | up | Molecular Function: DNA binding (GO:0003677);; | -- | -- | Homeobox domain | Uncharacterized protein OS=Glycine | Transcription | PREDICTED: WUSCHEL-related homeobox 1-like [Cucumis sativus] |
| 7 | Csa1G043150 | 1.96E-05 | 1.290651 | up | Cellular Component: cytoplasm (GO:0005737);; Molecular | -- | General function prediction only | Aldo/keto reductase family | Predicted protein GN=POPTRDRAF | General function prediction only | PREDICTED: aldo-keto reductase family 4 member C9-like, partial [Cucumis sativus] |
| 8 | Csa1G050380 | 0.018696 | 1.487238 | up | Molecular Function: argininosuccinate synthase activity (G( | Alanine, aspartate an | Amino acid transport and metabolism | Arginosuccinate synthase | Uncharacterized protein OS=Glycine | Amino acid transport and metabolism | PREDICTED: argininosuccinate synthase, chloroplastic-like [Cucumis sativus] |
| 9 | Csa1G058090 | 0.025365 | 1.336535 | up | -- | -- | General function prediction only | Domain of unknown function;; Ankyrin repeats (3 copies);; Ankyri | Uncharacterized protein GN=Bra027: | Function unknown | PREDICTED: ankyrin repeat-containing protein At5g02620-like [Cucumis sativus] |
| 10 | Csa1G058660 | 0.027776 | 2.23 | up | -- | -- | General function prediction only | Ankyrin repeats (3 copies);; Ankyrin repeat;; Ankyrin repeat;; Ank | Putative uncharacterized protein GN= | Function unknown | PREDICTED: uncharacterized protein LOC101223751, partial [Cucumis sativus] |
| 11 | Csa1G064740 | 1.08E-06 | 1.394361 | up | Molecular Function: protein kinase activity (GO:0004672);; | -- | -- | Kinase/pyrophosphorylase | Putative uncharacterized protein GN= | General function prediction only | PREDICTED: probable pyruvate, phosphate dikinase regulatory protein, chloroplastic-like [Cucumis sativus] |
| 12 | Csa1G073640 | 0.001795 | 2.030324 | up | Cellular Component: chloroplast (GO:0009507);; Cellular ( | -- | -- | Protein of unknown function (DUF_B2219);; Common central dom | Polyphenol oxidase GN=PPO3 OS=P | Function unknown | PREDICTED: polyphenol oxidase, chloroplastic-like [Cucumis sativus] |
| 13 | Csa1G073740 | 0.003449 | 1.760135 | up | -- | -- | -- | CBS domain | Putative uncharacterized protein GN= | Function unknown | PREDICTED: CBS domain-containing protein CBSX5-like [Cucumis sativus] |
| 14 | Csa1G096090 | 0.026825 | 1.045164 | up | Molecular Function: oxidoreductase activity, acting on CH- | -- | -- | Berberine and berberine like | Predicted protein GN=POPTRDRAF | Energy production and conversion | PREDICTED: reticuline oxidase-like protein-like [Cucumis sativus] |
| 15 | Csa1G097700 | 0.008206 | 1.020415 | up | Biological Process: maltose metabolic process (GO:00000: | -- | -- | -- | Predicted protein GN=POPTRDRAF | Function unknown | PREDICTED: uncharacterized protein LOC101218484 [Cucumis sativus] |
| 16 | Csa1G152500 | 1.12E-15 | 1.981699 | up | Cellular Component: chloroplast (GO:0009507);; Molecula | -- | -- | CBS domain | Putative uncharacterized protein GN= | Function unknown | PREDICTED: CBS domain-containing protein CBSX1, chloroplastic-like [Cucumis sativus] |
| 17 | Csa1G153560 | 0.011823 | 2.455989 | up | Cellular Component: cytoplasmic membrane-bounded vesic | -- | -- | Collagen triple helix repeat (20 copies) | Uncharacterized protein GN=PGSC0( | Extracellular structures | hypothetical protein CC1G_13073 [Coprinopsis cinerea okayama7#130] |
| 18 | Csa1G163160 | 0.034797 | 1.572608 | up | Molecular Function: electron carrier activity (GO:0009055 | Photosynthesis (ko00 | -- | 2Fe-2S iron-sulfur cluster binding domain | Putative uncharacterized protein GN= | General function prediction only | PREDICTED: ferredoxin-3, chloroplastic-like isoform 2 [Cucumis sativus] |
| 19 | Csa1G248110 | 0.003673 | 2.046869 | up | Biological Process: carbohydrate transport (GO:0008643);; | -- | General function prediction only | Sugar (and other) transporter;; Major Facilitator Superfamily | Uncharacterized protein GN=PRUPE_ | General function prediction only | PREDICTED: probable polyol transporter 6-like [Cucumis sativus] |
| 20 | Csa1G250130 | 1.90E-07 | 1.144552 | up | Molecular Function: nucleotide binding (GO:0000166);; Ce | Porphyrin and chloro | General function prediction only | short chain dehydrogenase;; Enoyl-(Acyl carrier protein) reductase | Short-chain dehydrogenase, putative ( | Secondary metabolites biosynthesis, transp | PREDICTED: probable chlorophyll(ide) b reductase NYC1, chloroplastic-like [Cucumis sativus] |
| 21 | Csa1G267230 | 0.000216 | 1.037045 | up | Molecular Function: hydrolase activity, hydrolyzing O-glyco | Amino sugar and nuc | Carbohydrate transport and metabolism | Glycosyl hydrolases family 18 | Predicted protein GN=POPTRDRAF | Carbohydrate transport and metabolism | PREDICTED: acidic mammalian chitinase-like [Cucumis sativus] |
| 22 | Csa1G278500 | 0.004117 | -1.05948 | down | -- | -- | -- | -- | Putative uncharacterized protein GN= | -- | PREDICTED: uncharacterized LOC101204868 [Cucumis sativus] |
| 23 | Csa1G420360 | 0.004887 | -2.1321 | down | Molecular Function: protein serine/threonine kinase activity | -- | Function unknown | Cysteine-rich secretory protein family | Uncharacterized protein OS=Lotus ja | Function unknown | PREDICTED: pathogenesis-related protein PR-1-like [Cucumis sativus] |
| 24 | Csa1G524640 | 1.29E-06 | 1.3052 | up | Molecular Function: iron ion binding (GO:0005506);; Mole | Carotenoid biosynthe | Secondary metabolites biosynthesis, tra | Cytochrome P450 | Uncharacterized protein GN=PRUPE_ | Secondary metabolites biosynthesis, transp | PREDICTED: abscisic acid 8'-hydroxylase 2-like [Cucumis sativus] |
| 25 | Csa1G526840 | 0.004717 | -1.21069 | down | Molecular Function: transferase activity, transferring hexosy | -- | Carbohydrate transport and metabolism | UDP-glucoronosyl and UDP-glucosyl transferase | Uncharacterized protein GN=PRUPE_ | Energy production and conversion;; Carbol | PREDICTED: UDP-glycosyltransferase 76E2-like [Cucumis sativus] |
| 26 | Csa1G527870 | 0.008361 | -1.23536 | down | Cellular Component: cytoplasm (GO:0005737);; Biological | Protein processing in | Posttranslational modification, protein t | Hsp20/alpha crystallin family | HSP18.2A OS=Citrullus lanatus (Wa | Posttranslational modification, protein turn | PREDICTED: 18.5 kDa class I heat shock protein-like [Cucumis sativus] |
| 27 | Csa1G528590 | 2.55E-13 | 1.978537 | up | Biological Process: maltose metabolic process (GO:00000: | Thiamine metabolism | -- | ThiC family;; ThiC-associated domain | Uncharacterized protein GN=PRUPE_ | Coenzyme transport and metabolism | PREDICTED: phosphomethylpyrimidine synthase, chloroplastic-like [Cucumis sativus] |
| 28 | Csa1G533490 | 0.02282 | -2.20625 | down | Molecular Function: protein serine/threonine phosphatase a | -- | Signal transduction mechanisms;; Gener | Calcineurin-like phosphoesterase | Putative uncharacterized protein GN= | Signal transduction mechanisms | PREDICTED: uncharacterized protein At1g18480-like [Cucumis sativus] |
| 29 | Csa1G575010 | 0.031476 | -1.64522 | down | Cellular Component: cytoplasm (GO:0005737);; Biological | Pentose and glucuron | -- | Pectinesterase | Pectinesterase GN=RCOM_1049330 | Carbohydrate transport and metabolism | PREDICTED: probable pectinesterase 53-like [Cucumis sativus] |
| 30 | Csa1G596520 | 1.96E-06 | 1.151463 | up | Cellular Component: extracellular region (GO:0005576);; C | -- | Posttranslational modification, protein t | Papain family cysteine protease;; Cathepsin propeptide inhibitor d | Predicted protein GN=POPTRDRAF | Posttranslational modification, protein turn | PREDICTED: thiol protease aleurain-like [Cucumis sativus] |
| 31 | Csa1G602540 | 5.28E-06 | -2.42285 | down | Molecular Function: copper ion binding (GO:0005507);; Ce | Ascorbate and aldara | Secondary metabolites biosynthesis, tra | Multicopper oxidase;; Multicopper oxidase;; Multicopper oxidase | Ascorbate oxidase AO1 OS=Cucumis | Secondary metabolites biosynthesis, transp | PREDICTED: LOW QUALITY PROTEIN: L-ascorbate oxidase-like [Cucumis sativus] |
| 32 | Csa1G612920 | 4.33E-05 | 1.18218 | up | Molecular Function: sequence-specific DNA binding transc | -- | Chromatin structure and dynamics;; Sig | Cupin-like domain;; Cupin superfamily protein;; JmjC domain, hyc | Putative uncharacterized protein GN= | Chromatin structure and dynamics;; Signal | PREDICTED: lysine-specific demethylase 8-like [Cucumis sativus] |
| 33 | Csa1G615150 | 4.37E-13 | 1.536472 | up | -- | -- | Carbohydrate transport and metabolism | Protein of unknown function, DUF604;; Fringe-like | Uncharacterized protein (Fragment) C | Carbohydrate transport and metabolism | PREDICTED: uncharacterized protein LOC101213989 [Cucumis sativus] |
| 34 | Csa1G627390 | 3.98E-06 | 1.862739 | up | Molecular Function: catalytic activity (GO:0003824);; Mol | Cyanoamino acid me | General function prediction only | GMC oxidoreductase;; GMC oxidoreductase | Uncharacterized protein (Fragment) C | Carbohydrate transport and metabolism | PREDICTED: (R)-mandelonitrile lyase 1-like [Cucumis sativus] |
| 35 | Csa1G649550 | 9.74E-11 | 1.271208 | up | Molecular Function: iron ion binding (GO:0005506);; Mole | -- | Secondary metabolites biosynthesis, tra | Cytochrome P450 | Cytochrome P450 GN=CYP716A9 O | Secondary metabolites biosynthesis, transp | PREDICTED: cytochrome P450 716B1-like [Cucumis sativus] |
| 36 | Csa1G660200 | 2.99E-22 | 2.31785 | up | Biological Process: defense response to nematode (GO:000 | -- | -- | Glycosyl hydrolases family 17 | Beta-1,3-glucanase GN=Gns3 OS=Pr | General function prediction only | PREDICTED: glucan endo-1,3-beta-glucosidase, basic isoform-like [Cucumis sativus] |
| 37 | Csa1G662790 | 7.86E-11 | 1.724434 | up | Cellular Component: extracellular region (GO:0005576);; M | -- | -- | Cupin;; Cupin domain | Rhicadhesin receptor, putative GN=R | General function prediction only | PREDICTED: germin-like protein subfamily 1 member 17-like [Cucumis sativus] |
| 38 | Csa2G000920 | 0.00284 | 1.578831 | up | -- | -- | Posttranslational modification, protein t | Ring finger domain;; RING-H2 zinc finger;; Zinc finger, C3HC4 ty | Putative uncharacterized protein GN= | Posttranslational modification, protein turn | PREDICTED: E3 ubiquitin-protein ligase RING1-like [Cucumis sativus] |
| 39 | Csa2G004700 | 0.04512 | 1.053607 | up | Cellular Component: mitochondrion (GO:0005739);; | -- | General function prediction only | PPR repeat;; PPR repeat family;; PPR repeat;; Pentatricopeptide re | Putative uncharacterized protein GN= | General function prediction only | PREDICTED: pentatricopeptide repeat-containing protein At4g38010-like [Cucumis sativus] |
| 40 | Csa2G004720 | 0.02173 | -2.07141 | down | Molecular Function: sequence-specific DNA binding transc | -- | Transcription | Multiprotein bridging factor 1;; Helix-turn-helix | Putative uncharacterized protein GN= | Transcription | PREDICTED: multiprotein-bridging factor 1c-like [Cucumis sativus] |
| 41 | Csa2G009550 | 1.91E-05 | -3.27275 | down | Cellular Component: nucleus (GO:0005634);; Biological Pr | -- | -- | Protein of unknown function (DUF640) | Putative uncharacterized protein GN= | Function unknown | PREDICTED: uncharacterized protein LOC101222316 [Cucumis sativus] |
| 42 | Csa2G059200 | 0.030931 | 1.185267 | up | Molecular Function: sequence-specific DNA binding transc | -- | -- | AUX/IAA family | Transcription factor, putative GN=RC | Transcription | PREDICTED: auxin-responsive protein IAA33-like [Cucumis sativus] |
| 43 | Csa2G061530 | 0.022961 | -1.58614 | down | Molecular Function: 1-phosphatidylinositol binding (GO:00 | -- | Signal transduction mechanisms;; Intrac | ANTH domain;; ENTH domain | Uncharacterized protein GN=PRUPE_ | Nucleotide transport and metabolism | PREDICTED: LOW QUALITY PROTEIN: putative clathrin assembly protein At4g25940-like [Cucumis sativus] |
| 44 | Csa2G108600 | 0.000254 | -1.13068 | down | Biological Process: response to external stimulus (GO:0009 | -- | -- | -- | Putative uncharacterized protein GN= | Signal transduction mechanisms | PREDICTED: protein PHYTOCHROME KINASE SUBSTRATE 1-like [Cucumis sativus] |
| 45 | Csa2G140360 | 2.34E-14 | 2.159112 | up | -- | -- | -- | Nucleotide-diphospho-sugar transferase | Uncharacterized protein OS=Glycine | Function unknown | PREDICTED: uncharacterized protein At1g28695-like [Cucumis sativus] |
| 46 | Csa2G215510 | 0.001512 | -1.03146 | down | Molecular Function: calcium ion binding (GO:0005509);; B | Plant-pathogen intera | Signal transduction mechanisms | EF hand;; EF-hand domain pair;; EF-hand domain;; EF hand;; EF-h | Calmodulin, putative GN=RCOM_09 | Signal transduction mechanisms | PREDICTED: calmodulin-like protein 8-like [Cucumis sativus] |
| 47 | Csa2G225320 | 3.31E-08 | -4.19319 | down | Biological Process: cellular process (GO:0009987);; Biolo | Plant hormone signal | -- | AUX/IAA family | Predicted protein GN=POPTRDRAF | Transcription | PREDICTED: auxin-induced protein AUX28-like [Cucumis sativus] |
| 48 | Csa2G264590 | 0.002886 | -2.16303 | down | Biological Process: amino acid transmembrane transport (G | Plant hormone signal | Amino acid transport and metabolism | Transmembrane amino acid transporter protein | Uncharacterized protein GN=PRUPE_ | Amino acid transport and metabolism | PREDICTED: auxin transporter-like protein 5-like [Cucumis sativus] |
| 49 | Csa2G308350 | 0.04512 | 1.592416 | up | Cellular Component: cytoplasm (GO:0005737);; Cellular C | -- | -- | WD domain, G-beta repeat | WD-repeat protein, putative GN=RC( | General function prediction only | PREDICTED: uncharacterized WD repeat-containing protein C2A9.03-like [Cucumis sativus] |
| 50 | Csa2G348790 | 4.58E-16 | -1.58135 | down | Molecular Function: polygalacturonase activity (GO:00046 | -- | -- | Glycosyl hydrolases family 28;; Pectate lyase superfamily protein | Putative uncharacterized protein GN= | General function prediction only | PREDICTED: polygalacturonase At1g48100-like [Cucumis sativus] |
| 51 | Csa2G349090 | 2.53E-05 | 1.001471 | up | -- | -- | -- | AP2 domain | Putative uncharacterized protein GN= | Function unknown | PREDICTED: ethylene-responsive transcription factor ESR1-like [Cucumis sativus] |
| 52 | Csa2G352950 | 4.17E-05 | 1.181865 | up | -- | -- | -- | -- | -- | -- | PREDICTED: uncharacterized protein LOC101203486 [Cucumis sativus] |
| 53 | Csa2G356090 | 2.77E-06 | -1.34603 | down | -- | -- | -- | Leucine rich repeat;; Leucine Rich Repeat | LRR-family protein OS=Cucumis mel | Function unknown | PREDICTED: uncharacterized protein At4g06744-like [Cucumis sativus] |
| 54 | Csa2G357340 | 0.002159 | 1.109453 | up | -- | -- | Signal transduction mechanisms | EF-hand domain pair;; EF hand;; EF hand;; EF-hand domain;; EF-h | Putative uncharacterized protein GN= | Signal transduction mechanisms | conserved hypothetical protein [Ricinus communis] |
| 55 | Csa2G359890 | 0.003921 | 2.394233 | up | #N/A | #N/A | #N/A | #N/A | #N/A | #N/A | #N/A |
| 56 | Csa2G359930 | 0.007618 | 1.109445 | up | -- | -- | Signal transduction mechanisms | EF hand;; EF-hand domain;; EF-hand domain pair;; EF-hand doma | Uncharacterized protein GN=Solyc04 | Signal transduction mechanisms | PREDICTED: probable calcium-binding protein CML27-like [Cucumis sativus] |
| 57 | Csa2G359940 | 0.003473 | 1.311092 | up | -- | -- | Signal transduction mechanisms | EF hand;; EF-hand domain;; EF-hand domain pair;; EF hand;; EF-h | Putative uncharacterized protein GN= | Signal transduction mechanisms | PREDICTED: probable calcium-binding protein CML10-like [Cucumis sativus] |
| 58 | Csa2G363550 | 7.77E-19 | -3.18806 | down | Molecular Function: transporter activity (GO:0005215);; Bi | -- | -- | -- | Glutamate receptor OS=Capsella rub | -- | PREDICTED: glutamate receptor 2.9-like [Cucumis sativus] |
| 59 | Csa2G363570 | 6.51E-07 | 1.135036 | up | Cellular Component: vacuole (GO:0005773);; Biological P | -- | Posttranslational modification, protein t | Papain family cysteine protease;; Cathepsin propeptide inhibitor d | JHL18I08.12 protein GN=JHL18I08. | Posttranslational modification, protein turn | PREDICTED: cysteine proteinase RD19a-like [Cucumis sativus] |
| 60 | Csa2G381830 | 0.018141 | -1.11613 | down | Molecular Function: transcription cofactor activity (GO:00 | -- | -- | -- | Putative uncharacterized protein GN= | Function unknown | PREDICTED: uncharacterized protein LOC101206923 [Cucumis sativus] |
| 61 | Csa2G382440 | 0.007333 | 1.01936 | up | Cellular Component: vacuole (GO:0005773);; Cellular Con | -- | Intracellular trafficking, secretion, and v | SPFH domain / Band 7 family | Putative uncharacterized protein GN= | Intracellular trafficking, secretion, and vesi | PREDICTED: LOW QUALITY PROTEIN: flotillin-like protein 4-like [Cucumis sativus] |
| 62 | Csa2G385070 | 0.02479 | 1.278837 | up | Molecular Function: phosphatidate cytidylyltransferase acti | -- | Function unknown | Cytidylyltransferase family | Uncharacterized protein GN=PRUPE_ | Function unknown | PREDICTED: probable phytol kinase 1, chloroplastic-like [Cucumis sativus] |
| 63 | Csa2G401440 | 0.003012 | 2.422028 | up | Biological Process: response to hypoxia (GO:0001666);; C | Starch and sucrose m | Cell wall/membrane/envelope biogenes | Glycosyl transferases group 1;; Sucrose-6F-phosphate phosphohy | Sucrose-phosphate synthase GN=sps | Cell wall/membrane/envelope biogenesis | PREDICTED: probable sucrose-phosphate synthase 4-like [Cucumis sativus] |
| 64 | Csa2G416240 | 0.002162 | 1.498065 | up | Molecular Function: transferase activity (GO:0016740);; | -- | -- | Cellulose synthase | Predicted protein GN=POPTRDRAF | Function unknown | PREDICTED: cellulose synthase-like protein H1-like [Cucumis sativus] |
| 65 | Csa2G429030 | 0.000748 | 2.241728 | up | Molecular Function: transferase activity, transferring acyl g | -- | -- | Transferase family | Putative alcohol acyl-transferases GN | General function prediction only | PREDICTED: benzyl alcohol O-benzoyltransferase-like [Cucumis sativus] |
| 66 | Csa3G002800 | 0.024528 | 1.491096 | up | Molecular Function: naringenin 3-dioxygenase activity (GO | Flavonoid biosynthes | Secondary metabolites biosynthesis, tra | non-haem dioxygenase in morphine synthesis N-terminal;; 2OG-Fe | Flavanone 3-hydroxylase OS=Cucum | Secondary metabolites biosynthesis, transp | PREDICTED: naringenin,2-oxoglutarate 3-dioxygenase-like [Cucumis sativus] |
| 67 | Csa3G019340 | 0.017993 | -1.974 | down | Cellular Component: vacuole (GO:0005773);; Cellular Con | Fatty acid elongation | Lipid transport and metabolism;; Posttr | Palmitoyl protein thioesterase | Putative uncharacterized protein GN= | Carbohydrate transport and metabolism | PREDICTED: palmitoyl-protein thioesterase 1-like, partial [Vitis vinifera] |
| 68 | Csa3G026690 | 4.62E-07 | 1.690788 | up | -- | -- | -- | Hydrophobic seed protein | Putative uncharacterized protein (Fra | General function prediction only | PREDICTED: uncharacterized LOC101221023 [Cucumis sativus] |
| 69 | Csa3G027190 | 2.67E-06 | 1.473229 | up | Biological Process: response to auxin (GO:0009733);; Biol | -- | -- | Hydrophobic seed protein | Putative cell wall-plasma membrane | General function prediction only | PREDICTED: uncharacterized protein LOC101209814 [Cucumis sativus] |
| 70 | Csa3G027200 | 2.55E-13 | 1.417129 | up | Biological Process: single-organism cellular process (GO:( | -- | -- | Hydrophobic seed protein | HyPRP2 GN=HyPRP2 OS=Gossypiu | General function prediction only | PREDICTED: 14 kDa proline-rich protein DC2.15-like [Cucumis sativus] |
| 71 | Csa3G047730 | 0.004931 | 1.054877 | up | Molecular Function: O-methyltransferase activity (GO:0008 | Phenylpropanoid bio | General function prediction only | O-methyltransferase | Uncharacterized protein GN=PRUPE_ | General function prediction only | PREDICTED: caffeic acid 3-O-methyltransferase-like [Cucumis sativus] |
| 72 | Csa3G065250 | 2.11E-13 | 2.771559 | up | -- | -- | -- | -- | Putative uncharacterized protein GN= | -- | glycoside hydrolase family 76 protein [Bipolaris maydis C5] |
| 73 | Csa3G077660 | 2.41E-06 | -1.51076 | down | Molecular Function: 1-acylglycerol-3-phosphate O-acyltran | -- | Lipid transport and metabolism | Acyltransferase | Predicted protein GN=POPTRDRAF | Lipid transport and metabolism | PREDICTED: 1-acyl-sn-glycerol-3-phosphate acyltransferase-like [Cucumis sativus] |
| 74 | Csa3G078800 | 0.000106 | 2.184085 | up | Biological Process: maltose metabolic process (GO:00000: | Thiamine metabolism | General function prediction only | Thi4 family;; FAD dependent oxidoreductase;; FAD binding doma | Uncharacterized protein GN=PRUPE_ | General function prediction only | PREDICTED: thiamine thiazole synthase, chloroplastic-like [Cucumis sativus] |
| 75 | Csa3G081380 | 0.015933 | 1.470931 | up | Cellular Component: chloroplast stroma (GO:0009570);; | -- | -- | -- | Uncharacterized protein GN=MTR_2 | Function unknown | PREDICTED: uncharacterized protein LOC101210146 [Cucumis sativus] |
| 76 | Csa3G116790 | 0.009024 | 1.047814 | up | Molecular Function: copper ion binding (GO:0005507);; Ce | -- | -- | Plastocyanin-like domain | Uclacyanin-2, putative GN=RCOM_( | General function prediction only | PREDICTED: blue copper protein-like [Cucumis sativus] |
| 77 | Csa3G124800 | 2.74E-07 | 1.108693 | up | Cellular Component: integral component of membrane (GO: | -- | Carbohydrate transport and metabolism | UAA transporter family;; Triose-phosphate Transporter family | Uncharacterized protein OS=Glycine | Carbohydrate transport and metabolism | PREDICTED: UDP-galactose/UDP-glucose transporter 4-like [Cucumis sativus] |
| 78 | Csa3G127230 | 4.16E-05 | -1.15846 | down | Cellular Component: plant-type vacuole (GO:0000325);; M | -- | Secondary metabolites biosynthesis, tra | ABC transporter transmembrane region;; ABC transporter;; P-loop | Uncharacterized protein GN=Bra0034 | Defense mechanisms | PREDICTED: ABC transporter C family member 9-like [Cucumis sativus] |
| 79 | Csa3G134750 | 0.000251 | 1.183023 | up | Biological Process: response to wounding (GO:0009611);; | -- | Amino acid transport and metabolism | POT family;; Major Facilitator Superfamily | Uncharacterized protein GN=PRUPE_ | Inorganic ion transport and metabolism | PREDICTED: LOW QUALITY PROTEIN: peptide transporter PTR3-A-like [Cucumis sativus] |
| 80 | Csa3G142400 | 0.015739 | 1.42971 | up | Molecular Function: serine-type endopeptidase inhibitor act | -- | -- | Potato inhibitor I family | Protease inhibitor GN=MTR_1g0753 | Function unknown | PREDICTED: protease inhibitor HPI-like [Cucumis sativus] |
| 81 | Csa3G146330 | 0.015838 | 2.838231 | up | -- | -- | -- | Protein of unknown function (DUF4005);; IQ calmodulin-binding r | Putative uncharacterized protein GN= | Function unknown | PREDICTED: uncharacterized protein LOC101202972 [Cucumis sativus] |
| 82 | Csa3G149960 | 0.008386 | 1.386978 | up | Molecular Function: transporter activity (GO:0005215);; Bi | -- | Carbohydrate transport and metabolism | Major intrinsic protein | Uncharacterized protein GN=PRUPE_ | Carbohydrate transport and metabolism | PREDICTED: probable aquaporin NIP7-1-like [Cucumis sativus] |
| 83 | Csa3G164460 | 3.08E-05 | -1.04484 | down | -- | -- | -- | Protein of unknown function (DUF581) | Putative uncharacterized protein GN= | Function unknown | PREDICTED: uncharacterized protein LOC101206584 [Cucumis sativus] |
| 84 | Csa3G164560 | 0.000155 | 2.680573 | up | Molecular Function: hydrolase activity (GO:0016787);; Mo | -- | -- | Glycosyl hydrolases family 28 | Putative uncharacterized protein GN= | General function prediction only | PREDICTED: exopolygalacturonase-like [Cucumis sativus] |
| 85 | Csa3G167360 | 4.33E-05 | 1.384824 | up | Molecular Function: carboxylic ester hydrolase activity (G( | -- | -- | GDSL-like Lipase/Acylhydrolase | Zinc finger protein, putative GN=RC( | General function prediction only | PREDICTED: GDSL esterase/lipase At4g16230-like [Cucumis sativus] |
| 86 | Csa3G181960 | 0.017723 | 1.017221 | up | -- | -- | -- | -- | Uncharacterized protein GN=PRUPE_ | Function unknown | PREDICTED: uncharacterized protein LOC101217506 [Cucumis sativus] |
| 87 | Csa3G183920 | 0.000185 | -1.29112 | down | Molecular Function: iron ion binding (GO:0005506);; Biolo | Carotenoid biosynthe | -- | Fatty acid hydroxylase superfamily | Chloroplast beta-carotene hydroxylas | Lipid transport and metabolism | PREDICTED: beta-carotene 3-hydroxylase, chloroplastic-like [Cucumis sativus] |
| 88 | Csa3G183960 | 1.62E-07 | 1.085963 | up | Molecular Function: DNA binding (GO:0003677);; Molecu | -- | Posttranslational modification, protein t | Myb-like DNA-binding domain | DNA binding protein, putative GN=R | Transcription | PREDICTED: transcription factor ASG4-like [Cucumis sativus] |
| 89 | Csa3G212490 | 4.16E-05 | 1.297469 | up | Biological Process: transition metal ion transport (GO:000( | -- | -- | WRKY DNA -binding domain | WRKY protein GN=WRKY19 OS=C | Transcription | uncharacterized protein LOC101215114 [Cucumis sativus] |
| 90 | Csa3G250900 | 1.37E-05 | 1.576299 | up | Biological Process: transcription, DNA-templated (GO:000 | -- | -- | Probable lipid transfer;; Protease inhibitor/seed storage/LTP famil | Putative uncharacterized protein GN= | General function prediction only | PREDICTED: lipid transfer-like protein VAS-like [Cucumis sativus] |
| 91 | Csa3G282490 | 0.006531 | -2.79285 | down | Molecular Function: binding (GO:0005488);; Biological Pr | -- | -- | Helix-loop-helix DNA-binding domain | Predicted protein GN=POPTRDRAF | General function prediction only | Basic helix-loop-helix DNA-binding superfamily protein, putative [Theobroma cacao] |
| 92 | Csa3G359120 | 0.002226 | -1.42178 | down | Molecular Function: ATP binding (GO:0005524);; Biologic | -- | Secondary metabolites biosynthesis, tra | ABC transporter;; ABC transporter transmembrane region;; AAA ( | Uncharacterized protein GN=PRUPE_ | Secondary metabolites biosynthesis, transp | PREDICTED: ABC transporter C family member 10-like [Cucumis sativus] |
| 93 | Csa3G414060 | 0.01573 | 1.035192 | up | Biological Process: maltose metabolic process (GO:00000: | Photosynthesis (ko00 | -- | Oxygen evolving enhancer protein 3 (PsbQ) | Uncharacterized protein GN=Bra039: | General function prediction only | PREDICTED: psbQ-like protein 2, chloroplastic-like [Cucumis sativus] |
| 94 | Csa3G446120 | 0.023896 | 1.469537 | up | Molecular Function: ATP binding (GO:0005524);; Cellular | -- | Secondary metabolites biosynthesis, tra | ABC-2 type transporter;; ABC transporter;; Plant PDR ABC transp | Pleiotropic drug resistance protein Gl | Secondary metabolites biosynthesis, transp | PREDICTED: ABC transporter G family member 36-like [Cucumis sativus] |
| 95 | Csa3G597290 | 0.01107 | 1.375501 | up | Biological Process: maltose metabolic process (GO:00000: | -- | Lipid transport and metabolism;; Gener | Methyltransferase domain;; Methyltransferase domain;; Methyltran | Predicted protein GN=POPTRDRAF | Lipid transport and metabolism | PREDICTED: uncharacterized protein LOC101221941 [Cucumis sativus] |
| 96 | Csa3G598910 | 0.001643 | 3.402296 | up | -- | -- | -- | Light regulated protein Lir1 | GA-like protein (Fragment) OS=Cucu | Function unknown | PREDICTED: light-regulated protein-like [Cucumis sativus] |
| 97 | Csa3G690300 | 0.011982 | 1.499385 | up | Biological Process: phosphatidylinositol biosynthetic proce | -- | -- | -- | Uncharacterized protein GN=PRUPE_ | Function unknown | RNA/RNP complex-1-interacting phosphatase, putative [Theobroma cacao] |
| 98 | Csa3G698510 | 0.001526 | 1.23222 | up | Molecular Function: monooxygenase activity (GO:0004497 | -- | Secondary metabolites biosynthesis, tra | Cytochrome P450 | Uncharacterized protein GN=PRUPE_ | Secondary metabolites biosynthesis, transp | PREDICTED: cytochrome P450 705A20-like [Cucumis sativus] |
| 99 | Csa3G733220 | 0.047063 | 1.429212 | up | -- | Protein processing in | Posttranslational modification, protein t | Hsp20/alpha crystallin family | HSP22.8 OS=Citrullus lanatus (Wate | Posttranslational modification, protein turn | PREDICTED: 18.5 kDa class I heat shock protein-like [Cucumis sativus] |
| 100 | Csa3G743950 | 0.026234 | 1.150985 | up | Biological Process: defense response (GO:0006952);; | -- | -- | Thaumatin family | Uncharacterized protein OS=Medicag | General function prediction only | PREDICTED: thaumatin-like protein 1-like [Cucumis sativus] |
| 101 | Csa3G750900 | 0.013387 | -1.42772 | down | Cellular Component: nucleolus (GO:0005730);; Cellular Co | -- | Translation, ribosomal structure and bio | Ribosomal protein L24e | Putative uncharacterized protein GN= | Translation, ribosomal structure and bioge | PREDICTED: PAN domain-containing protein At5g03700-like [Cucumis sativus] |
| 102 | Csa3G760500 | 3.38E-08 | -1.85245 | down | -- | -- | -- | Probable lipid transfer;; Protease inhibitor/seed storage/LTP famil | Uncharacterized protein OS=Glycine | General function prediction only | PREDICTED: putative lipid-transfer protein DIR1-like [Cucumis sativus] |
| 103 | Csa3G777660 | 0.000101 | 1.173634 | up | Cellular Component: plasma membrane (GO:0005886);; Bi | -- | -- | Domain of unknown function (DUF588) | Uncharacterized protein GN=PRUPE_ | Function unknown | PREDICTED: casparian strip membrane protein RCOM_1259250-like [Cucumis sativus] |
| 104 | Csa3G837020 | 0.001882 | 1.402588 | up | #N/A | #N/A | #N/A | #N/A | #N/A | #N/A | #N/A |
| 105 | Csa3G838720 | 0.021022 | 2.743998 | up | Molecular Function: hydrolase activity, hydrolyzing O-glyco | Galactose metabolisr | -- | Raffinose synthase or seed imbibition protein Sip1 | Raffinose synthase OS=Cucumis sativ | General function prediction only | PREDICTED: probable galactinol--sucrose galactosyltransferase 5-like [Cucumis sativus] |
| 106 | Csa3G851720 | 0.006408 | -1.49402 | down | Molecular Function: microtubule motor activity (GO:00037 | -- | Cytoskeleton | Dynein light chain type 1 | Uncharacterized protein OS=Glycine | Cytoskeleton | PREDICTED: dynein light chain LC6, flagellar outer arm-like [Cucumis sativus] |
| 107 | Csa3G860260 | 1.08E-06 | 1.014363 | up | Molecular Function: DNA binding (GO:0003677);; Molecu | -- | -- | Bifunctional nuclease | Uncharacterized protein GN=PRUPE_ | General function prediction only | PREDICTED: bifunctional nuclease 1-like [Cucumis sativus] |
| 108 | Csa3G874380 | 0.004117 | -1.52677 | down | Cellular Component: cytosol (GO:0005829);; Biological Pr | -- | General function prediction only | Thioredoxin-like;; Thioredoxin-like domain;; AhpC/TSA family;; T | Uncharacterized protein GN=PRUPE_ | Posttranslational modification, protein turn | PREDICTED: probable nucleoredoxin 2-like [Cucumis sativus] |
| 109 | Csa3G895620 | 6.59E-06 | -1.19326 | down | Molecular Function: nucleotide binding (GO:0000166);; Mo | -- | -- | Leucine Rich repeats (2 copies);; Leucine rich repeat;; Leucine Ri | Uncharacterized protein GN=PRUPE_ | Signal transduction mechanisms | PREDICTED: probably inactive leucine-rich repeat receptor-like protein kinase At2g25790-like [Cucumis sativus] |
| 110 | Csa3G895910 | 0.048169 | -1.08901 | down | Cellular Component: mitochondrion (GO:0005739);; Biolog | -- | General function prediction only | PPR repeat family;; PPR repeat;; Pentatricopeptide repeat domain; | Uncharacterized protein GN=PRUPE_ | General function prediction only | PREDICTED: pentatricopeptide repeat-containing protein At1g31920-like [Cucumis sativus] |
| 111 | Csa4G001830 | 0.000155 | 1.457346 | up | Molecular Function: epoxide hydrolase activity (GO:00043 | -- | Lipid transport and metabolism | Alpha/beta hydrolase family;; alpha/beta hydrolase fold;; Alpha/b | Uncharacterized protein GN=eh2 OS= | Lipid transport and metabolism | PREDICTED: bifunctional epoxide hydrolase 2-like [Cucumis sativus] |
| 112 | Csa4G003620 | 2.55E-13 | 3.191456 | up | Biological Process: response to molecule of fungal origin (( | -- | -- | Universal stress protein family | Putative uncharacterized protein GN= | Function unknown | PREDICTED: universal stress protein MJ0531-like [Cucumis sativus] |
| 113 | Csa4G003670 | 0.000401 | 1.816846 | up | Biological Process: response to light stimulus (GO:000941( | -- | -- | CP12 domain | Chloroplast protein 12 GN=CP12 OS | General function prediction only | PREDICTED: calvin cycle protein CP12-2, chloroplastic-like [Cucumis sativus] |
| 114 | Csa4G025120 | 0.000561 | 1.550559 | up | Molecular Function: transferase activity (GO:0016740);; | -- | -- | -- | Predicted protein GN=POPTRDRAF | -- | PREDICTED: uncharacterized protein LOC101203258 [Cucumis sativus] |
| 115 | Csa4G043850 | 0.004207 | -1.56835 | down | Molecular Function: sequence-specific DNA binding transc | -- | Transcription | Homeobox domain | Predicted protein GN=POPTRDRAF | Transcription | PREDICTED: homeobox-leucine zipper protein ATHB-21-like [Cucumis sativus] |
| 116 | Csa4G045010 | 0.000243 | 1.542168 | up | Molecular Function: peroxidase activity (GO:0004601);; Ce | Phenylalanine metabo | -- | Peroxidase | Peroxidase 72, putative GN=RCOM_ | Carbohydrate transport and metabolism | PREDICTED: peroxidase 72-like [Cucumis sativus] |
| 117 | Csa4G046790 | 1.40E-06 | 1.027701 | up | Biological Process: protein peptidyl-prolyl isomerization (( | -- | Posttranslational modification, protein t | PPIC-type PPIASE domain;; PPIC-type PPIASE domain | Uncharacterized protein GN=pin1 OS | Posttranslational modification, protein turn | PREDICTED: peptidyl-prolyl cis-trans isomerase Pin1-like [Cucumis sativus] |
| 118 | Csa4G047370 | 0.044723 | -1.54266 | down | Cellular Component: intracellular (GO:0005622);; Molecul | -- | -- | B-box zinc finger | Putative uncharacterized protein GN= | Function unknown | PREDICTED: probable salt tolerance-like protein At1g78600-like [Cucumis sativus] |
| 119 | Csa4G050120 | 0.00821 | 1.134549 | up | Molecular Function: sequence-specific DNA binding transc | Plant-pathogen intera | -- | WRKY DNA -binding domain | WRKY protein GN=WRKY26 OS=C | Transcription | WRKY transcription factor 22-like [Cucumis sativus] |
| 120 | Csa4G053280 | 0.009331 | 4.896036 | up | Cellular Component: membrane (GO:0016020);; Molecular | -- | -- | Cellulose synthase;; Glycosyl transferase family group 2 | Uncharacterized protein GN=PRUPE_ | Carbohydrate transport and metabolism | PREDICTED: cellulose synthase-like protein G3-like [Cucumis sativus] |
| 121 | Csa4G094530 | 6.41E-10 | -2.13519 | down | Molecular Function: serine-type endopeptidase activity (G( | -- | Signal transduction mechanisms | Rhomboid family | Putative uncharacterized protein GN= | Signal transduction mechanisms | PREDICTED: uncharacterized protein LOC101206746 [Cucumis sativus] |
| 122 | Csa4G112650 | 0.010675 | -1.64779 | down | Molecular Function: oxidoreductase activity, acting on pair( | -- | Secondary metabolites biosynthesis, tra | non-haem dioxygenase in morphine synthesis N-terminal;; 2OG-Fe | Uncharacterized protein GN=PRUPE_ | Secondary metabolites biosynthesis, transp | PREDICTED: flavonol synthase/flavanone 3-hydroxylase-like [Cucumis sativus] |
| 123 | Csa4G146240 | 0.012619 | 1.214731 | up | -- | -- | -- | Probable lipid transfer;; Protease inhibitor/seed storage/LTP famil | Non-specific lipid-transfer protein Gl | General function prediction only | Bifunctional inhibitor/lipid-transfer protein/seed storage 2S albumin superfamily protein, putative [Theobroma cacao] |
| 124 | Csa4G167980 | 0.007618 | -1.03888 | down | Cellular Component: cytoplasm (GO:0005737);; Biological | Galactose metabolisr | -- | Raffinose synthase or seed imbibition protein Sip1 | Alkaline alpha galactosidase GN=AC | General function prediction only | probable galactinol--sucrose galactosyltransferase 2-like [Cucumis sativus] |
| 125 | Csa4G189010 | 0.024145 | 1.746406 | up | Molecular Function: N-acyltransferase activity (GO:001641 | Phenylpropanoid bio | -- | Transferase family | Uncharacterized protein OS=Glycine | Function unknown | PREDICTED: spermidine hydroxycinnamoyl transferase-like [Cucumis sativus] |
| 126 | Csa4G260440 | 0.000236 | -1.63717 | down | Biological Process: regulation of mitotic cell cycle (GO:00 | -- | Function unknown | Neurochondrin | Uncharacterized protein GN=PRUPE_ | Function unknown | PREDICTED: neurochondrin-like [Cucumis sativus] |
| 127 | Csa4G285740 | 0.001408 | 2.311453 | up | Molecular Function: peroxidase activity (GO:0004601);; M | Phenylalanine metabo | -- | Peroxidase | Peroxidase (Precursor) GN=pre-pero | Carbohydrate transport and metabolism | PREDICTED: peroxidase 2-like [Cucumis sativus] |
| 128 | Csa4G287560 | 3.22E-05 | -3.76892 | down | Biological Process: fatty acid biosynthetic process (GO:00( | Linoleic acid metabo | -- | Lipoxygenase | Uncharacterized protein OS=Zea may | Amino acid transport and metabolism | PREDICTED: lipoxygenase 2, chloroplastic-like [Cucumis sativus] |
| 129 | Csa4G293000 | 0.013387 | 1.135836 | up | Cellular Component: mitochondrion (GO:0005739);; | -- | General function prediction only | PPR repeat family;; Pentatricopeptide repeat domain;; PPR repeat; | Putative uncharacterized protein GN= | General function prediction only | PREDICTED: pentatricopeptide repeat-containing protein At5g02830, chloroplastic-like [Cucumis sativus] |
| 130 | Csa4G295490 | 0.008501 | -1.42134 | down | -- | -- | -- | -- | Predicted protein GN=POPTRDRAF | Function unknown | PREDICTED: uncharacterized protein LOC101214483 [Cucumis sativus] |
| 131 | Csa4G303690 | 0.036688 | -1.47375 | down | Biological Process: response to stress (GO:0006950);; Biol | Glutathione metaboli | Posttranslational modification, protein t | Glutathione S-transferase, N-terminal domain;; Glutathione S-tran | Putative uncharacterized protein GN= | Posttranslational modification, protein turn | PREDICTED: probable glutathione S-transferase-like [Cucumis sativus] |
| 132 | Csa4G508490 | 0.001474 | 2.72694 | up | #N/A | #N/A | #N/A | #N/A | #N/A | #N/A | #N/A |
| 133 | Csa4G614200 | 0.018696 | 1.658767 | up | -- | -- | -- | AAA domain;; AAA domain;; Zeta toxin | Predicted protein GN=POPTRDRAF | Function unknown | predicted protein [Populus trichocarpa] |
| 134 | Csa4G645920 | 2.47E-05 | 2.084278 | up | Molecular Function: phosphoprotein phosphatase activity (( | -- | Signal transduction mechanisms | Protein phosphatase 2C;; Protein phosphatase 2C | Putative uncharacterized protein GN= | Signal transduction mechanisms | PREDICTED: probable protein phosphatase 2C 72-like [Cucumis sativus] |
| 135 | Csa4G648590 | 0.00035 | 1.292572 | up | -- | -- | -- | Protein of unknown function (DUF1677) | Predicted protein GN=POPTRDRAF | Function unknown | PREDICTED: uncharacterized protein LOC101216724 isoform 2 [Cucumis sativus] |
| 136 | Csa4G658600 | 8.53E-06 | 1.734474 | up | Molecular Function: catalase activity (GO:0004096);; Cellu | Tryptophan metaboli | Inorganic ion transport and metabolism | Catalase;; Catalase-related immune-responsive | Catalase (Fragment) GN=CAT OS=P | Inorganic ion transport and metabolism | catalase, partial [Populus x beijingensis] |
| 137 | Csa5G021300 | 1.27E-14 | 1.50901 | up | Molecular Function: core promoter binding (GO:0001047); | Circadian rhythm - pl | -- | Galactose oxidase, central domain;; Galactose oxidase, central do | Uncharacterized protein OS=Glycine | General function prediction only | PREDICTED: adagio protein 3-like [Cucumis sativus] |
| 138 | Csa5G092930 | 3.82E-12 | 1.995878 | up | Molecular Function: binding (GO:0005488);; Cellular Com | -- | General function prediction only | Zinc finger, C2H2 type | Predicted protein GN=POPTRDRAF | General function prediction only | PREDICTED: LOW QUALITY PROTEIN: zinc finger protein NUTCRACKER-like [Cucumis sativus] |
| 139 | Csa5G153010 | 0.024333 | -1.2384 | down | Molecular Function: iron ion binding (GO:0005506);; Biolo | Cutin, suberine and w | Secondary metabolites biosynthesis, tra | Cytochrome P450 | Putative uncharacterized protein GN= | Secondary metabolites biosynthesis, transp | PREDICTED: cytochrome P450 86A2-like [Cucumis sativus] |
| 140 | Csa5G168900 | 0.006417 | 1.372941 | up | Molecular Function: protein kinase activity (GO:0004672);; | -- | -- | Protein kinase domain;; Protein tyrosine kinase;; Leucine rich repe | Predicted protein GN=POPTRDRAF | Signal transduction mechanisms | PREDICTED: probable LRR receptor-like serine/threonine-protein kinase At4g08850-like [Cucumis sativus] |
| 141 | Csa5G168910 | 0.000283 | 2.326954 | up | #N/A | #N/A | #N/A | #N/A | #N/A | #N/A | #N/A |
| 142 | Csa5G175710 | 0.001106 | 1.73284 | up | Molecular Function: protein binding (GO:0005515);; Cellu | -- | Posttranslational modification, protein t | DnaJ domain;; 4Fe-4S single cluster domain;; 4Fe-4S single cluste | Putative uncharacterized protein GN= | Posttranslational modification, protein turn | PREDICTED: uncharacterized protein LOC101205271 [Cucumis sativus] |
| 143 | Csa5G184310 | 0.012505 | -3.3345 | down | Molecular Function: kinase activity (GO:0016301);; Biolog | -- | Function unknown | Regulator of chromosome condensation (RCC1) repeat;; Regulator | Putative uncharacterized protein GN= | Function unknown | PREDICTED: E3 ubiquitin-protein ligase HERC2-like [Cucumis sativus] |
| 144 | Csa5G187870 | 3.82E-19 | 2.382944 | up | Molecular Function: serine-type endopeptidase activity (G( | -- | -- | Subtilase family;; Peptidase inhibitor I9;; PA domain | Cucumisin OS=Siraitia grosvenorii (N | Posttranslational modification, protein turn | PREDICTED: cucumisin-like [Cucumis sativus] |
| 145 | Csa5G212090 | 0.008088 | 2.857705 | up | Molecular Function: acid phosphatase activity (GO:000399 | Glycolysis / Glucone | General function prediction only | Histidine phosphatase superfamily (branch 2) | Uncharacterized protein GN=PRUPE_ | General function prediction only | PREDICTED: multiple inositol polyphosphate phosphatase 1-like [Cucumis sativus] |
| 146 | Csa5G266880 | 7.72E-06 | 1.045364 | up | Cellular Component: chloroplast (GO:0009507);; Biologica | -- | -- | Pathogenesis-related protein Bet v I family | Uncharacterized protein GN=PRUPE_ | Function unknown | PREDICTED: MLP-like protein 28-like [Cucumis sativus] |
| 147 | Csa5G341040 | 0.000251 | -1.4333 | down | -- | -- | Posttranslational modification, protein t | Myb-like DNA-binding domain;; Myb-like DNA-binding domain | Putative uncharacterized protein GN= | Transcription | PREDICTED: uncharacterized protein LOC101212264 [Cucumis sativus] |
| 148 | Csa5G514500 | 0.000146 | -2.01861 | down | Molecular Function: ATP binding (GO:0005524);; Biologic | Spliceosome (ko030 | Posttranslational modification, protein t | Hsp70 protein;; MreB/Mbl protein;; Hydantoinase/oxoprolinase;; | Putative uncharacterized protein GN= | Posttranslational modification, protein turn | PREDICTED: heat shock cognate 70 kDa protein-like isoform 1 [Cucumis sativus] |
| 149 | Csa5G571570 | 0.015107 | 1.264487 | up | Cellular Component: nucleus (GO:0005634);; Cellular Com | -- | Posttranslational modification, protein t | DnaJ domain | Uncharacterized protein GN=PRUPE_ | Posttranslational modification, protein turn | PREDICTED: chaperone protein DnaJ-like [Cucumis sativus] |
| 150 | Csa5G585950 | 1.80E-06 | 1.033268 | up | Biological Process: response to superoxide (GO:0000303); | ABC transporters (k | Secondary metabolites biosynthesis, tra | ABC transporter transmembrane region;; ABC transporter;; RecF/ | Multidrug/pheromone exporter, MDR | Secondary metabolites biosynthesis, transp | PREDICTED: ABC transporter B family member 4-like [Cucumis sativus] |
| 151 | Csa5G589260 | 2.08E-11 | 1.481169 | up | Molecular Function: cysteine synthase activity (GO:000412 | Cysteine and methior | Amino acid transport and metabolism | Pyridoxal-phosphate dependent enzyme | Cysteine synthase GN=RCOM_0723: | Amino acid transport and metabolism | PREDICTED: cysteine synthase, chloroplastic/chromoplastic-like [Cucumis sativus] |
| 152 | Csa5G594450 | 0.011982 | 2.364679 | up | Molecular Function: monooxygenase activity (GO:0004497 | -- | Secondary metabolites biosynthesis, tra | Cytochrome P450 | Putative uncharacterized protein GN= | Secondary metabolites biosynthesis, transp | PREDICTED: cytochrome P450 71B37-like [Cucumis sativus] |
| 153 | Csa5G598700 | 0.000245 | 1.233044 | up | Cellular Component: chloroplast thylakoid membrane (GO:( | -- | -- | -- | Uncharacterized protein GN=PRUPE_ | Function unknown | PREDICTED: uncharacterized protein LOC101221356 [Cucumis sativus] |
| 154 | Csa5G599830 | 1.40E-08 | 1.318641 | up | Molecular Function: protein histidine kinase activity (GO:0 | -- | -- | Pyruvate phosphate dikinase, PEP/pyruvate binding domain | Uncharacterized protein GN=PRUPE_ | General function prediction only | PREDICTED: alpha-glucan water dikinase, chloroplastic-like [Cucumis sativus] |
| 155 | Csa5G622530 | 0.009386 | 1.07906 | up | Biological Process: ER to Golgi vesicle-mediated transport | -- | -- | Nodulin-like;; Major Facilitator Superfamily | Putative uncharacterized protein GN= | General function prediction only | PREDICTED: uncharacterized protein LOC101205503 [Cucumis sativus] |
| 156 | Csa5G622660 | 0.000164 | 1.177132 | up | Cellular Component: vacuolar membrane (GO:0005774);; ( | -- | Amino acid transport and metabolism | Transmembrane amino acid transporter protein | Uncharacterized protein GN=PRUPE_ | Amino acid transport and metabolism | PREDICTED: vacuolar amino acid transporter 1-like [Cucumis sativus] |
| 157 | Csa5G623810 | 0.00252 | 1.833079 | up | Cellular Component: plasma membrane (GO:0005886);; Bi | -- | Amino acid transport and metabolism | POT family;; Major Facilitator Superfamily | Uncharacterized protein OS=Glycine | Carbohydrate transport and metabolism | PREDICTED: probable peptide/nitrate transporter At3g54450-like [Cucumis sativus] |
| 158 | Csa5G630820 | 0.032827 | 1.053245 | up | Biological Process: regulation of transcription, DNA-templ | -- | Transcription | Homeobox associated leucine zipper;; Homeobox domain | Putative uncharacterized protein GN= | Transcription | PREDICTED: homeobox-leucine zipper protein HAT5-like [Cucumis sativus] |
| 159 | Csa6G003390 | 3.17E-05 | 1.224773 | up | Cellular Component: extracellular region (GO:0005576);; ( | -- | General function prediction only | Glycosyl transferase family 90 | Putative uncharacterized protein GN= | General function prediction only | PREDICTED: protein O-glucosyltransferase 1-like [Cucumis sativus] |
| 160 | Csa6G004590 | 0.000136 | 1.036375 | up | Cellular Component: chloroplast stroma (GO:0009570);; Bi | -- | -- | -- | Putative uncharacterized protein GN= | Function unknown | PREDICTED: uncharacterized protein LOC101221288 [Cucumis sativus] |
| 161 | Csa6G008070 | 0.048044 | 1.20316 | up | Biological Process: amino acid transmembrane transport (G | -- | Amino acid transport and metabolism | Transmembrane amino acid transporter protein;; Tryptophan/tyros | Uncharacterized protein OS=Glycine | Amino acid transport and metabolism | PREDICTED: lysine histidine transporter 1-like [Cucumis sativus] |
| 162 | Csa6G040540 | 9.74E-11 | 1.477637 | up | Molecular Function: iron ion binding (GO:0005506);; Cellu | Flavonoid biosynthes | Secondary metabolites biosynthesis, tra | 2OG-Fe(II) oxygenase superfamily;; non-haem dioxygenase in mor | Uncharacterized protein OS=Glycine | Coenzyme transport and metabolism | PREDICTED: flavonol synthase/flavanone 3-hydroxylase-like [Cucumis sativus] |
| 163 | Csa6G088010 | 5.28E-06 | 1.105583 | up | -- | -- | -- | TPR repeat;; Tetratricopeptide repeat;; Tetratricopeptide repeat;; | Putative uncharacterized protein GN= | Function unknown | Tetratricopeptide repeat-like superfamily protein, putative isoform 3 [Theobroma cacao] |
| 164 | Csa6G095280 | 0.031449 | -5.02034 | down | -- | -- | -- | -- | MADS-box protein 15 GN=PMADS1 | -- | unnamed protein product [Vitis vinifera] |
| 165 | Csa6G124120 | 0.014718 | -1.06246 | down | Biological Process: response to light stimulus (GO:000941( | Plant hormone signal | -- | GH3 auxin-responsive promoter | Putative uncharacterized protein OS= | General function prediction only | PREDICTED: probable indole-3-acetic acid-amido synthetase GH3.5-like [Cucumis sativus] |
| 166 | Csa6G147580 | 0.000748 | -1.38175 | down | Biological Process: metabolic process (GO:0008152);; Bio | -- | Carbohydrate transport and metabolism | UDP-glucoronosyl and UDP-glucosyl transferase | Uncharacterized protein GN=PRUPE_ | Energy production and conversion;; Carbol | PREDICTED: UDP-glycosyltransferase 86A2-like [Cucumis sativus] |
| 167 | Csa6G155030 | 0.003862 | -1.02849 | down | Molecular Function: L-tyrosine:2-oxoglutarate aminotransfe | Ubiquinone and other | Amino acid transport and metabolism | Aminotransferase class I and II;; Beta-eliminating lyase | Putative uncharacterized protein GN= | Amino acid transport and metabolism | PREDICTED: tyrosine aminotransferase-like [Cucumis sativus] |
| 168 | Csa6G188010 | 6.43E-10 | 1.217672 | up | Cellular Component: cytosol (GO:0005829);; Molecular Fu | Starch and sucrose m | Carbohydrate transport and metabolism | Carbohydrate phosphorylase | Phosphorylase GN=CmPhoH1 OS=C | Carbohydrate transport and metabolism | PREDICTED: alpha-glucan phosphorylase, H isozyme-like [Cucumis sativus] |
| 169 | Csa6G188030 | 0.012785 | 1.914984 | up | #N/A | #N/A | #N/A | #N/A | #N/A | #N/A | #N/A |
| 170 | Csa6G410040 | 0.002654 | 1.097886 | up | -- | -- | -- | Protein of unknown function (DUF3049) | Putative uncharacterized protein GN= | Function unknown | conserved hypothetical protein [Ricinus communis] |
| 171 | Csa6G416850 | 0.016551 | 1.413085 | up | Molecular Function: catalytic activity (GO:0003824);; Biol | -- | -- | -- | Pherophorin-dz1 protein (Precursor) ( | Function unknown | pherophorin-dz1 protein [Volvox carteri f. nagariensis] |
| 172 | Csa6G418400 | 4.58E-16 | -2.33056 | down | Molecular Function: transferase activity, transferring glycos | -- | Carbohydrate transport and metabolism | -- | Uncharacterized protein GN=PRUPE_ | Energy production and conversion;; Carbol | PREDICTED: UDP-glycosyltransferase 74E2-like [Cucumis sativus] |
| 173 | Csa6G418940 | 0.000164 | -2.12427 | down | Biological Process: metabolic process (GO:0008152);; Bio | -- | Carbohydrate transport and metabolism | UDP-glucoronosyl and UDP-glucosyl transferase | UDP-glucosyltransferase, putative GN | Energy production and conversion;; Carbol | PREDICTED: UDP-glycosyltransferase 74E2-like [Cucumis sativus] |
| 174 | Csa6G421760 | 1.84E-05 | 1.022315 | up | -- | Protein processing in | Posttranslational modification, protein t | Hsp20/alpha crystallin family | HSP27.8 OS=Citrullus lanatus (Wate | Posttranslational modification, protein turn | PREDICTED: uncharacterized protein LOC101211982 isoform 1 [Cucumis sativus] |
| 175 | Csa6G423470 | 8.12E-08 | 1.847902 | up | Biological Process: metabolic process (GO:0008152);; Mo | Glycine, serine and t | Secondary metabolites biosynthesis, tra | Copper amine oxidase, enzyme domain;; Copper amine oxidase, N | Amine oxidase GN=POPTRDRAFT_ | Secondary metabolites biosynthesis, transp | PREDICTED: primary amine oxidase-like [Cucumis sativus] |
| 176 | Csa6G424000 | 3.82E-14 | -1.50081 | down | Molecular Function: iron ion binding (GO:0005506);; Cellu | Linoleic acid metabo | -- | Lipoxygenase | Lipoxygenase GN=lox9 OS=Cucumis | Function unknown | probable linoleate 9S-lipoxygenase 5-like [Cucumis sativus] |
| 177 | Csa6G424540 | 1.45E-06 | 2.006419 | up | Molecular Function: high-affinity hydrogen:glucose symport | -- | General function prediction only | Sugar (and other) transporter | Hexose carrier (Fragment) GN=Hex9 | General function prediction only | PREDICTED: sugar transport protein 13-like [Cucumis sativus] |
| 178 | Csa6G425130 | 0.013127 | 1.745962 | up | -- | -- | -- | Protein of unknown function (DUF3049) | Putative uncharacterized protein GN= | Function unknown | conserved hypothetical protein [Ricinus communis] |
| 179 | Csa6G425740 | 0.000262 | 1.275671 | up | Biological Process: response to stress (GO:0006950);; Biol | -- | -- | -- | Putative uncharacterized protein GN= | General function prediction only | PREDICTED: probable F-box protein At1g60180-like, partial [Cucumis sativus] |
| 180 | Csa6G430740 | 1.47E-12 | 1.360352 | up | Cellular Component: endosome (GO:0005768);; Cellular C | -- | -- | -- | Uncharacterized protein GN=PRUPE_ | Function unknown | PREDICTED: uncharacterized protein LOC101207236 [Cucumis sativus] |
| 181 | Csa6G450350 | 0.01132 | -1.22751 | down | Cellular Component: Golgi apparatus (GO:0005794);; Biol | -- | -- | Glycosyltransferase like family 2;; Glycosyl transferase family gro | Uncharacterized protein OS=Glycine | General function prediction only | PREDICTED: glucomannan 4-beta-mannosyltransferase 9-like [Cucumis sativus] |
| 182 | Csa6G485160 | 0.001526 | 1.52716 | up | -- | -- | -- | EamA-like transporter family | Uncharacterized protein GN=PRUPE_ | Function unknown | PREDICTED: auxin-induced protein 5NG4-like [Cucumis sativus] |
| 183 | Csa6G490030 | 0.011982 | -1.03761 | down | Molecular Function: DNA binding (GO:0003677);; Molecu | -- | -- | Dof domain, zinc finger | Uncharacterized protein (Fragment) C | Transcription | PREDICTED: dof zinc finger protein DOF3.4-like [Cucumis sativus] |
| 184 | Csa6G499090 | 0.000141 | 1.054598 | up | Molecular Function: dicarboxylic acid transmembrane trans | -- | Energy production and conversion | Mitochondrial carrier protein | Putative uncharacterized protein OS= | Energy production and conversion | PREDICTED: mitochondrial uncoupling protein 5-like [Cucumis sativus] |
| 185 | Csa6G499180 | 4.45E-13 | -1.97648 | down | Cellular Component: plasma membrane (GO:0005886);; Bi | -- | General function prediction only | MatE | Predicted protein GN=POPTRDRAF | Function unknown | PREDICTED: LOW QUALITY PROTEIN: protein TRANSPARENT TESTA 12-like [Cucumis sativus] |
| 186 | Csa6G500600 | 0.047063 | -2.42849 | down | Molecular Function: inositol-3-phosphate synthase activity ( | Inositol phosphate m | Lipid transport and metabolism | Myo-inositol-1-phosphate synthase;; Myo-inositol-1-phosphate sy | L-myo-inositol-1-phosphate synthase | Lipid transport and metabolism | PREDICTED: inositol-3-phosphate synthase-like [Cucumis sativus] |
| 187 | Csa6G504660 | 0.005146 | 1.346684 | up | Cellular Component: Golgi apparatus (GO:0005794);; Cell | -- | -- | Putative S-adenosyl-L-methionine-dependent methyltransferase;; M | ATP binding protein, putative GN=R( | General function prediction only | PREDICTED: probable methyltransferase PMT27-like [Cucumis sativus] |
| 188 | Csa6G507520 | 8.90E-07 | 2.412975 | up | Molecular Function: chitinase activity (GO:0004568);; Cell | Amino sugar and nuc | General function prediction only | Chitinase class I;; Chitin recognition protein | Endochitinase MCHT-2 GN=mcht-2 ( | Carbohydrate transport and metabolism | PREDICTED: basic endochitinase A-like [Cucumis sativus] |
| 189 | Csa6G513450 | 9.66E-06 | 1.175241 | up | Biological Process: response to cold (GO:0009409);; Biolo | -- | -- | RbcX protein | Putative uncharacterized protein GN= | Function unknown | PREDICTED: uncharacterized protein LOC101222925 [Cucumis sativus] |
| 190 | Csa6G513580 | 8.76E-08 | 1.299683 | up | Cellular Component: peroxisomal membrane (GO:0005778 | Peroxisome (ko0414( | General function prediction only | Mpv17 / PMP22 family | Uncharacterized protein OS=Glycine | General function prediction only | PREDICTED: peroxisomal membrane protein PMP22-like [Cucumis sativus] |
| 191 | Csa6G521010 | 0.033452 | 1.171865 | up | Molecular Function: ATP binding (GO:0005524);; Biologic | ABC transporters (k | Secondary metabolites biosynthesis, tra | ABC transporter transmembrane region;; ABC transporter;; AAA ( | Uncharacterized protein GN=PRUPE_ | Secondary metabolites biosynthesis, transp | PREDICTED: ABC transporter B family member 9-like [Cucumis sativus] |
| 192 | Csa7G002520 | 3.31E-08 | 1.602523 | up | Cellular Component: cytoplasm (GO:0005737);; Molecular | -- | -- | NmrA-like family;; NADH(P)-binding;; NAD dependent epimeras | Isoflavone reductase-like protein 5 G | General function prediction only | PREDICTED: isoflavone reductase homolog [Cucumis sativus] |
| 193 | Csa7G004020 | 4.28E-34 | 4.249564 | up | Biological Process: response to cadmium ion (GO:004668( | -- | -- | NmrA-like family;; NADH(P)-binding;; 3-beta hydroxysteroid deh | Isoflavone reductase related protein ( | General function prediction only | PREDICTED: isoflavone reductase homolog [Cucumis sativus] |
| 194 | Csa7G029980 | 6.82E-41 | 5.360783 | up | Biological Process: maltose metabolic process (GO:00000: | -- | -- | Protein kinase domain;; PAS domain;; Protein tyrosine kinase;; PA | Uncharacterized protein OS=Glycine | Signal transduction mechanisms | PREDICTED: phototropin-2-like [Cucumis sativus] |
| 195 | Csa7G044230 | 0.00256 | 1.131959 | up | Cellular Component: nucleus (GO:0005634);; | -- | -- | Protein of unknown function (DUF2781) | Uncharacterized protein OS=Glycine | Function unknown | PREDICTED: transmembrane protein 97-like [Cucumis sativus] |
| 196 | Csa7G044880 | 0.00051 | 2.689676 | up | Biological Process: polysaccharide catabolic process (GO: | -- | -- | Glycosyl hydrolase family 14 | Beta-amylase OS=Castanea crenata ( | General function prediction only | PREDICTED: beta-amylase-like [Cucumis sativus] |
| 197 | Csa7G047420 | 5.02E-12 | 1.373253 | up | Cellular Component: extracellular region (GO:0005576);; E | -- | -- | Pollen allergen;; Rare lipoprotein A (RlpA)-like double-psi beta-l | Putative uncharacterized protein PtrE | General function prediction only | PREDICTED: expansin-B3-like [Cucumis sativus] |
| 198 | Csa7G051380 | 0.011091 | 1.996484 | up | Biological Process: response to abiotic stimulus (GO:0009( | -- | Carbohydrate transport and metabolism | UDP-glucoronosyl and UDP-glucosyl transferase | Putative uncharacterized protein GN= | Energy production and conversion;; Carbol | PREDICTED: UDP-glycosyltransferase 74E2-like [Cucumis sativus] |
| 199 | Csa7G061710 | 0.047434 | 3.10695 | up | Molecular Function: peroxidase activity (GO:0004601);; Ce | Phenylalanine metabo | -- | Peroxidase | Uncharacterized protein GN=PGSC0( | Carbohydrate transport and metabolism | PREDICTED: peroxidase N1-like [Cucumis sativus] |
| 200 | Csa7G066240 | 0.045923 | 1.868421 | up | -- | -- | -- | Phloem filament protein PP1 | Uncharacterized protein OS=Musa ac | -- | Cystatin/monellin superfamily protein, putative [Theobroma cacao] |
| 201 | Csa7G071700 | 0.013888 | -1.13609 | down | Molecular Function: alcohol dehydrogenase activity, zinc-d | Phenylpropanoid bio | Secondary metabolites biosynthesis, tra | Alcohol dehydrogenase GroES-like domain;; Zinc-binding dehydro | Cinnamyl alcohol dehydrogenase GN | Secondary metabolites biosynthesis, transp | PREDICTED: cinnamyl alcohol dehydrogenase 1-like [Cucumis sativus] |
| 202 | Csa7G098730 | 0.034638 | 1.345846 | up | -- | -- | -- | -- | Putative uncharacterized protein GN= | -- | conserved hypothetical protein [Ricinus communis] |
| 203 | Csa7G168060 | 0.008367 | 2.500628 | up | Molecular Function: DNA binding (GO:0003677);; Molecu | -- | Posttranslational modification, protein t | Myb-like DNA-binding domain | DNA binding protein, putative GN=R | Transcription | PREDICTED: transcription factor RADIALIS-like [Cucumis sativus] |
| 204 | Csa7G293740 | 5.28E-06 | 1.03064 | up | Cellular Component: nucleoplasm (GO:0005654);; Biologic | Circadian rhythm - pl | -- | -- | Uncharacterized protein GN=PRUPE_ | Function unknown | PREDICTED: protein GIGANTEA-like [Cucumis sativus] |
| 205 | Csa7G312940 | 1.79E-05 | 1.227605 | up | Molecular Function: structural constituent of cell wall (GO: | -- | -- | Extensin-like region | Uncharacterized protein GN=Bra025( | General function prediction only | PREDICTED: extensin-3-like [Cucumis sativus] |
| 206 | Csa7G343870 | 0.002487 | 1.688134 | up | Molecular Function: hydrolase activity (GO:0016787);; Bio | Pentose and glucuron | -- | Pectinesterase;; Plant invertase/pectin methylesterase inhibitor | Pectinesterase GN=PME2 OS=Gossy | Carbohydrate transport and metabolism | PREDICTED: pectinesterase 3-like [Cucumis sativus] |
| 207 | Csa7G393460 | 0.031476 | 1.975007 | up | Molecular Function: catalytic activity (GO:0003824);; | -- | General function prediction only | Phenazine biosynthesis-like protein | Uncharacterized protein GN=PRUPE_ | General function prediction only | PREDICTED: uncharacterized isomerase BH0283-like [Cucumis sativus] |
| 208 | Csa7G398090 | 0.045118 | 1.111348 | up | Biological Process: MAPK cascade (GO:0000165);; Biolog | -- | -- | Late embryogenesis abundant protein | Uncharacterized protein GN=PRUPE_ | Function unknown | PREDICTED: indole-3-acetic acid-induced protein ARG2-like [Cucumis sativus] |
| 209 | Csa7G414480 | 0.024415 | 1.298508 | up | Molecular Function: oxidoreductase activity (GO:0016491) | -- | General function prediction only | Enoyl-(Acyl carrier protein) reductase;; short chain dehydrogenase | Predicted protein GN=POPTRDRAF | Secondary metabolites biosynthesis, transp | PREDICTED: short-chain dehydrogenase reductase 3b-like isoform 1 [Cucumis sativus] |
| 210 | Csa7G429530 | 0.004113 | 2.523442 | up | Cellular Component: nucleus (GO:0005634);; | -- | -- | -- | Putative uncharacterized protein GN= | Function unknown | PREDICTED: uncharacterized protein LOC101208199 [Cucumis sativus] |
| 211 | Csa7G432450 | 0.007485 | 1.026928 | up | Cellular Component: chloroplast stroma (GO:0009570);; Ce | -- | -- | -- | Putative uncharacterized protein GN= | Function unknown | PREDICTED: uncharacterized protein LOC101207565 [Cucumis sativus] |
| 212 | Csa7G451390 | 0.034535 | 3.651004 | up | Molecular Function: iron ion binding (GO:0005506);; Mole | Brassinosteroid bios | Secondary metabolites biosynthesis, tra | Cytochrome P450 | Cytochrome p450 OS=Cucumis melo | Secondary metabolites biosynthesis, transp | cytochrome p450 [Cucumis melo subsp. melo] |
| 213 | CsaUNG014620 | 0.034837 | 1.719403 | up | Cellular Component: nucleus (GO:0005634);; Biological Pr | -- | -- | Pathogenesis-related protein Bet v I family;; Polyketide cyclase / c | Uncharacterized protein GN=PRUPE_ | Function unknown | PREDICTED: MLP-like protein 28-like [Cucumis sativus] |
| 214 | CsaUNG033660 | 0.006906 | 1.057018 | up | Cellular Component: chloroplast (GO:0009507);; Biologica | -- | -- | Protein of unknown function (DUF3110) | Putative uncharacterized protein GN= | Function unknown | PREDICTED: uncharacterized protein LOC101211866 [Cucumis sativus] |
| 215 | cucumber_newGene_[illegible] | 0.00252 | 1.925811 | up | -- | Cyanoamino acid me | Secondary metabolites biosynthesis, tra | Cytochrome P450 | Isoleucine N-monooxygenase 2 GN=( | Secondary metabolites biosynthesis, transp | PREDICTED: isoleucine N-monooxygenase 1 [Cucumis melo] |
| 216 | cucumber_newGene_[illegible] | 4.28E-07 | -2.77315 | down | -- | -- | General function prediction only | zinc-binding in reverse transcriptase | -- | General function prediction only | PREDICTED: putative ribonuclease H protein At1g65750-like [Cucumis sativus] |
| 217 | cucumber_newGene_[illegible] | 0.04955 | 1.190379 | up | Cellular Component: vacuole (GO:0005773);; Biological P | Steroid biosynthesis | Lipid transport and metabolism | Prenyltransferase and squalene oxidase repeat;; Prenyltransferase- | Cucurbitadienol synthase GN=CPQ C | Lipid transport and metabolism | oxidosqualene cyclase [Cucumis sativus] |
| 218 | cucumber_newGene_[illegible] | 8.65E-05 | 1.018179 | up | -- | alpha-Linolenic acid | Energy production and conversion;; Ger | NADH:flavin oxidoreductase / NADH oxidase family | 12-oxophytodienoate reductase 1 GN | Energy production and conversion | PREDICTED: 12-oxophytodienoate reductase 1-like [Cucumis sativus] |
| 219 | cucumber_newGene_[illegible] | 0.028614 | -2.34168 | down | Biological Process: carbohydrate metabolic process (GO:0( | -- | -- | Glycosyl hydrolase family 9;; Glycosyl hydrolase family 9 | Endoglucanase 1 (Precursor) GN=CE | General function prediction only | PREDICTED: endoglucanase 1-like [Cucumis sativus] |
| 220 | cucumber_newGene_[illegible] | 2.53E-05 | -1.68235 | down | -- | -- | -- | -- | -- | -- | PREDICTED: calcium-transporting ATPase 12, plasma membrane-type-like [Cucumis melo] |
| 221 | cucumber_newGene_[illegible] | 0.027077 | 1.703294 | up | -- | -- | -- | Stress up-regulated Nod 19 | -- | General function prediction only | hypothetical protein Csa_1G024275 [Cucumis sativus] |
| 222 | cucumber_newGene_[illegible] | 1.14E-05 | Inf | up | Molecular Function: oxidoreductase activity (GO:0016491) | -- | General function prediction only | Aldo/keto reductase family | NADP-dependent D-sorbitol-6-phosp | General function prediction only | PREDICTED: NADP-dependent D-sorbitol-6-phosphate dehydrogenase-like [Cucumis sativus] |
| 223 | cucumber_newGene_[illegible] | 0.006491 | 1.439282 | up | -- | -- | -- | Pathogenesis-related protein Bet v I family | MLP-like protein 329 GN=MLP329 ( | Function unknown | PREDICTED: MLP-like protein 329-like [Cucumis sativus] |
| 224 | cucumber_newGene_[illegible] | 8.53E-06 | -1.10215 | down | #N/A | #N/A | #N/A | #N/A | #N/A | #N/A | #N/A |

**Supplemental Table S2. RNA-Seq analysis of differentially expressed genes involved in organ development in the *mf* mutant compared with the wild-type**

| Gene ID | $log_2FC$ | FDR | Putative functions |
|---|---|---|---|
| Csa3G895620 | -1.19 | 6.59E-06 | Leucine-rich receptor-like protein kinase family |
| Csa4G043850 | -1.57 | 4.21 E-03 | Homeobox-leucine zipper protein family |
| Csa6G095280 | -5.02 | 3.14 E-02 | Agamous-like MADS-box protein |
| Csa5G341040 | -1.43 | 2.51 E-04 | Myb-like transcription factor REVEILLE 1-like |
| Csa4G043850 | -1.57 | 4.21 E-03 | homeobox-leucine zipper protein ATHB-21-like |
| Csa5G630820 | 1.05 | 3.28 E-02 | homeobox-leucine zipper protein HAT5-like |
| Csa2G352950 | 1.18 | 4.17E-05 | transcription factor RAX3-like |
| Csa3G183960 | 1.09 | 1.62E-07 | transcription factor ASG4-like |
| Csa7G168060 | 2.5 | 8.37 E-03 | transcription factor RADIALIS-like |

**Supplemental Table S3. Cucumber genes analyzed in this study and their putative functions**

| Name in this study | Gene ID | Annotation in CuGenDB (https://cucurbitgenomics.org/) |
|---|---|---|
| CsWOX1 | Csa1G042780 | WUSCHEL-related homeobox; contains IPR009057 (Homeodomain-like) |
| CsPID1 | Csa1G537400 | Protein kinase; contains IPR011009 (Protein kinase-like domain) |
| CsPID2 | Csa2G006100 | Kinase family protein; contains IPR011009 (Protein kinase-like domain) |
| CsPID2-like | Csa4G064100 | Serine/threonine protein kinase, putative; contains IPR011009 (Protein kinase-like domain) |
| CsPIN1 | Csa1G042820 | Auxin efflux carrier; contains IPR004776 (Auxin efflux carrier) |
| CsPIN2 | Csa5G284520 | Auxin efflux carrier; contains IPR004776 (Auxin efflux carrier) |
| CsPIN3 | Csa1G025070 | Auxin efflux carrier; contains IPR004776 (Auxin efflux carrier) |
| CsPIN4 | Csa4G430820 | Auxin efflux carrier; contains IPR004776 (Auxin efflux carrier) |
| CsPIN5 | Csa1G427480 | Auxin efflux carrier protein; contains IPR004776 (Auxin efflux carrier) |
| CsPIN6 | Csa5G576590 | Auxin efflux carrier; contains IPR004776 (Auxin efflux carrier) |
| CsAUX1 | Csa4G308640 | Auxin transporter-like protein; contains IPR013057 (Amino acid transporter, transmembrane) |
| CsLAX1 | Csa3G731880 | Auxin transporter-like protein; contains IPR013057 (Amino acid transporter, transmembrane) |
| CsLAX2 | Csa7G010800 | Auxin transporter-like protein; contains IPR013057 (Amino acid transporter, transmembrane) |
| CsLAX3 | Csa5G374730 | Auxin transporter-like protein; contains IPR013057 (Amino acid transporter, transmembrane) |
| CsLAX4 | Csa2G264590 | Auxin transporter-like protein; contains IPR013057 (Amino acid transporter, transmembrane) |
| CsLAX5 | Csa5G201310 | Auxin transporter-like protein; contains IPR013057 (Amino acid transporter, transmembrane) |
| CsLAX6 | Csa6G011040 | Auxin transporter-like protein; contains IPR013057 (Amino acid transporter, transmembrane) |
| CsTCP2a | Csa6G524000 | TCP transcription factor; contains IPR005333 (Transcription factor, TCP) |
| CsTCP2b | Csa1G025920 | TB1-like TCP family transcription factor; contains IPR005333 (Transcription factor, TCP) |
| CsTCP4a | Csa4G088720 | Transcription factor; contains IPR005333 (Transcription factor, TCP) |
| CsTCP4b | Csa6G156050 | Transcription factor; contains IPR005333 (Transcription factor, TCP) |
| CsTCP4c | Csa4G628330 | Transcription factor; contains IPR005333 (Transcription factor, TCP) |
| CsTCP4d | Csa1G077150 | Transcription factor; contains IPR005333 (Transcription factor, TCP) |
| CsTCP5a | Csa4G132680 | TB1-like TCP family transcription factor; contains IPR005333 (Transcription factor, TCP) |
| CsTCP5b | Csa1G014310 | Transcription factor; contains IPR005333 (Transcription factor, TCP) |
| CsTCP5c | Csa1G039270 | TCP transcription factor; contains IPR005333 (Transcription factor, TCP) |

**Supplemental Table S4. The parameters of vein pattern in cucumber leaves of Fig. 5B**

| **Leaf** | **α Angle (°)** | **β Angle (°)** | **Total length of primary vein (cm)** | **primary vein density (cm / $cm^2$)** | **Total length of secondary vein (cm)** | **Number of secondary vein** | **Average length of secondary vein (cm)** | **Secondary vein density (cm / $cm^2$)** | **Leaf area ($cm^2$)** |
|---|---|---|---|---|---|---|---|---|---|
| AM218 | 57.7±1.2 | 91.8±1.3 | 40.2±1.8 | 0.62±0.04 | 71.4±2.8 | 26±0 | 2.75±0.11 | 1.11±0.05 | 64.5±3.6 |
| *rl* | 31.8±0.8** | 96.7±1.5** | 29.2±1.6** | 0.44±0.02** | 97.5±4.5** | 18±0** | 5.42±0.25** | 1.46±0.10** | 66.8±3.7 |
| *mf* | 30.0±0.9** | 67.2±1.6** | 38.9±1.9 | 1.14±0.09** | 55.2±3.1** | 27±1** | 2.03±0.12** | 1.62±0.10** | 34.2±2.5** |
| *CsWOX1* -OE | 41.2±0.8** | 87.9±1.3** | 19.6±1.2** | 0.76±0.07** | 34.8±2.2** | 22±0** | 1.58±0.10** | 0.76±0.07** | 25.9±2.5** |

Asterisks indicate a significant difference (t test, P < 0.01).

**Supplemental Table S5. Primer sequences used in this study**

| Primers | Gene ID[a] | Forward primer (5' to 3') | Reverse primer (5' to 3') |
|---|---|---|---|
| 1305.4-CsWOX1 | Csa1G042780 | ACGGGGGACTCTAGAGGATCCATGTGGATGATGGGTTACAAT | CTGGTCACCAATTCACACGTGTTAGTGGTGGTGGTGGTGATTCTTCAATGGTAGGAATTCA |
| BD-CsWOX1 | Csa1G042780 | ATGGCCATGGAGGCCGAATTCATGTGGATGATGGGTTACAATGAC | ATGCGGCCGCTGCAGGTCGACTCAATTCTTCAATGGTAGGAATTC |
| BD-CsWOX1N | Csa1G042780 | ATGGCCATGGAGGCCGAATTCATGTGGATGATGGGTTACAATGAC | ATGCGGCCGCTGCAGGTCGACACCACCACGGAGAGCAGCG |
| NE-CsWOX1 | Csa1G042780 | CCCAGGCCTACTAGTGGATCCATGTGGATGATGGGTTACAATGAC | CCCGGGAGCGGTACCCTCGAGTCAATTCTTCAATGGTAGGAATTC |
| NE-CsWOX1N | Csa1G042780 | CCCAGGCCTACTAGTGGATCCATGTGGATGATGGGTTACAATGAC | CCCGGGAGCGGTACCCTCGAGACCACCACGGAGAGCAGCG |
| AD-CsTCP4a | Csa4G088720 | GTGGGCATCGATACGGGATCCATATGGGAGAGAGCCACCGC | ACGATTCATCTGCAGCTCGAGTCAATGGCGAGAATCAGAGGA |
| AD-CsAP2 | Csa6G491020 | GTGGGCATCGATACGGGATCCATATGGGGTGCGACGCTTTC | ACGATTCATCTGCAGCTCGAGTCAAGAGGGTCTCATAAGACAATGA |
| CRISPR-AtWOX1, 3 | At3G18010, AT2G28610 | TCGAAGTAGTGATTGGCGCAGATTCCTTCAACGGGTTTTAGAGCTAGAAATAG | TTCTAGCTCTAAAACCGTTGAAGCCACAGGACTCCAATCTCTTAGTCGACTCTAC |
| pGREEN-P35-CsWOX1 | Csa1G042780 | GCCCAAGCTACGCGTCTCGAGATGTGGATGATGGGTTACAATGAC | TCCCCCGGGCTGCAGGAATTCATTCTTCAATGGTAGGAATTCAAAG |
| Check-HYG517 | –– | TCGTTATGTTTATCGGCACTTT | GATGTTGGCGACCTCGTATT |
| Check-eGFP427 | –– | TCCAGGAGCGCACCATCTT | TCGTCCATGCCGTGAGTGA |
| qCsActin2 | Csa6G484600 | ATTCTTGCATCTCTAAGTACCTTCC | CCAACTAAAGGGAAATAACTCACC |
| qCsWOX1 | Csa1G042780 | GGGGCAACAGATTGCAGGAC | GGGCAATGTTCATCATTAGTTGG |
| qCsCLE5 | Csa4G627800 | ATGGCTAATTGTAGTGTAAAGCTG | GATAAACTTCAACATTTCTTCCG |
| qCsCLE6 | Csa6G518900 | CTTAGCTTGTGGGTATGCCTGA | CCCTCGTTGAGGAATGCTTC |
| qCsPID1 | Csa1G537400 | TCATCCGTTTTTGCCTACACT | ACTTCCGCTGCATAAAACCT |
| qCsPID2 | Csa2G006100 | CACGCCTCCGACTCCATCTA | GTCCACCAATCCACTCCGC |
| qCsPID2-like | Csa4G064100 | GAGATTCTGGCTTCTTTGGACC | GCAACAGAGAAGCGGCTACC |
| qCsPIN1 | Csa1G042820 | GCTACAACAGGGGGCAATG | TCCTCTGTTTTCCCATTGGC |
| qCsPIN2 | Csa5G284520 | CTTTCAACTTTGCCGAATACG | CAGAATCTTCGCTCCACGAT |
| qCsPIN3 | Csa1G025070 | ATCATCGTTCTGGTGGTGCT | TGAACCATTAAGCTGCCAGA |
| qCsPIN4 | Csa4G430820 | GAATGTACGGCGACTCCACT | ACAATCTCCCCTGCAGTATCC |
| qCsPIN5 | Csa1G427480 | GAGCTTCGAATCTCACCGGA | CGTCGCCTTGAAAACTATTGG |
| qCsPIN6 | Csa5G576590 | TCATTTCATTCCGGTGGCAT | GGCAAAAGTAGCAACAGAGTTCC |
| qCsAUX1 | Csa4G308640 | GGAAAGGAGGAAGGGGAAGA | AGGCAGAGTCAAAAGCACCTG |
| qCsLAX1 | Csa3G731880 | TTGTTTAAGGGCTATTGCGAG | CAGCATATGAGCCATAGCAGG |
| qCsLAX2 | Csa7G010800 | TTTGGGTGTTGGTAGTTGGC | GCCGTAGCATTGAACGATTG |
| qCsLAX3 | Csa5G374730 | TTGGACCGATCAACTCGACA | TTCACTGAGTACAGCCCTGCC |
| qCsLAX4 | Csa2G264590 | GTCTACATCATCCCCGCCC | CACCAAAGCCAAACCCTACA |
| qCsLAX5 | Csa5G201310 | GGGCTTGTTGTGCCACTACT | GAATGCTTCACACCCTCGAC |
| qCsLAX6 | Csa6G011040 | TCCCAACTGGGTATGCTCTC | ACCCATCCAGCACTTCAAAC |
| qCsTCP2a | Csa6G524000 | TCCAGAAACTCCGAAACAGC | CGCGAAAGAGACAAACCTGA |
| qCsTCP2b | Csa1G025920 | CATCGGTGGAGTTGGTGCTA | AGCGACAGAAAGCCGAACTC |
| qCsTCP4a | Csa4G088720 | AGGAGGCCACATTGTTCGTT | CAGCTTTACTGGGTCGGTCA |
| qCsTCP4b | Csa6G156050 | CACCAACTACCACAATGGGAA | TGAGCGGATAAACGAACACG |
| qCsTCP4c | Csa4G628330 | GCTGGAGGTGTGGATGGAGA | GCGTGTAACTGGACTGAAGGG |
| qCsTCP4d | Csa1G077150 | CACAGTTCCAGTCTTACCCACTTC | TCCCACATCGGAAAGAAAGG |
| qCsTCP5a | Csa4G132680 | TTCTCAGCCCTTTTGAATAATGT | TGAGATGATTGAAGGTATGAAGGT |
| qCsTCP5b | Csa1G014310 | CCCTACCTCCTCCGTTTATGC | TCAATCCACTTCCCTTTCGTC |
| qCsTCP5c | Csa1G039270 | CATTCTGATATTGGAGATGCTGC | GGGGCTGAATTAGTCTGATCG |
| CsWOX1-sp6 | Csa1G042780 | GATTTAGGTGACACTATAGaatGCTGTCGACAAATGGAATCTTCAGC | –– |
| CsWOX1-T7 | Csa1G042780 | TGTAATACGACTCACTATAGGGGAAAACAATGCAACAATTGCAAA | –– |

[a] The gene ID is based on 9930 V2.0.
